# Comparative sensitivity evaluation for 122 CE-marked rapid diagnostic tests for SARS-CoV-2 antigen, Germany, September 2020 to April 2021

**DOI:** 10.2807/1560-7917.ES.2021.26.44.2100441

**Published:** 2021-11-04

**Authors:** Heinrich Scheiblauer, Angela Filomena, Andreas Nitsche, Andreas Puyskens, Victor M Corman, Christian Drosten, Karin Zwirglmaier, Constanze Lange, Petra Emmerich, Michael Müller, Olivia Knauer, C Micha Nübling

**Affiliations:** 1 Testing Laboratory for In-vitro Diagnostic Medical Devices, Paul-Ehrlich-Institute, Langen, Germany; 2 Robert Koch Institute, Highly Pathogenic Viruses, Centre for Biological Threats and Special Pathogens, WHO Reference Laboratory for SARS-CoV-2 and WHO Collaborating Centre for Emerging Infections and Biological Threats, Robert Koch Institute, Berlin, Germany; 3 Charité – Universitätsmedizin Berlin, Institute of Virology and German Centre for Infection Research (DZIF), Associated Partner Site, Berlin, Germany; 4 Labor Berlin, Charité - Vivantes GmbH, Berlin, Germany; 5 Bundeswehr Institute of Microbiology, and German Centre for Infection Research (DZIF), Partner Site, Munich, Germany; 6 LADR Central Laboratory Dr. Kramer & Colleagues, Geesthacht, Germany; 7 Bernhard-Nocht Institute, Department of Virology, Hamburg, and Department of Tropical Medicine and Infectious Diseases, Center of Internal Medicine II, University of Rostock, Rostock, Germany; 8 MVZ Labor 28 GmbH, Berlin, Germany

**Keywords:** SARS-CoV-2 antigen, rapid diagnostic test, CE mark, COVID 19, sensitivity evaluation

## Abstract

**Introduction:**

Numerous CE-marked SARS-CoV-2 antigen rapid diagnostic tests (Ag RDT) are offered in Europe, several of them with unconfirmed quality claims.

**Aim:**

We performed an independent head-to-head evaluation of the sensitivity of SARS-CoV-2 Ag RDT offered in Germany.

**Methods:**

We addressed the sensitivity of 122 Ag RDT in direct comparison using a common evaluation panel comprised of 50 specimens. Minimum sensitivity of 75% for panel specimens with a PCR quantification cycle (Cq) ≤ 25 was used to identify Ag RDT eligible for reimbursement in the German healthcare system.

**Results:**

The sensitivity of different SARS-CoV-2 Ag RDT varied over a wide range. The sensitivity limit of 75% for panel members with Cq ≤ 25 was met by 96 of the 122 tests evaluated; 26 tests exhibited lower sensitivity, few of which failed completely. Some RDT exhibited high sensitivity, e.g. 97.5 % for Cq < 30.

**Conclusions:**

This comparative evaluation succeeded in distinguishing less sensitive from better performing Ag RDT. Most of the evaluated Ag RDT appeared to be suitable for fast identification of acute infections associated with high viral loads. Market access of SARS-CoV-2 Ag RDT should be based on minimal requirements for sensitivity and specificity.

## Introduction

A large number of antigen-detecting rapid diagnostic tests (Ag RDT) for severe acute respiratory syndrome coronavirus 2 (SARS-CoV-2) are available on the European market, both for professional use and as self-tests. Rapid tests are based on lateral flow immunochromatography using antibodies against SARS-CoV-2 proteins (antigens), present in respiratory tract specimens. By far most Ag RDT target the viral nucleoprotein, only very few assays work with spike protein detection. Viral variants of concern (VOC) contain mainly mutations in the gene encoding the spike protein, leaving the vast majority of SARS-CoV‑2 Ag RDT unaffected; however, the few SARS-CoV-2 Ag RDT based on spike protein detection should be checked at regular intervals for potential deficiencies. While PCR is still the gold standard for virus detection, there is increasing evidence that infectivity of respiratory secretions correlates with high viral loads present in the early phase of infection, e.g. before and 0–10 days after onset of symptoms. In addition to more complex and time-consuming PCR systems, Ag RDT allow rapid identification of acutely infected and potentially infectious individuals facilitating fast decisions on containment of virus spread, patient care, isolation and contact tracing [1,2]. Furthermore, Ag RDT may save limited reagents of more sensitive molecular diagnostics to serve other diagnostic needs, e.g. disease management or confirmation of Ag RDT reactive results.

In the European Union (EU), regulatory requirements for SARS-CoV-2 in vitro diagnostic medical devices (IVD) are defined by the IVD Directive 98/79/EC (IVDD) and have to be addressed by the manufacturer before access to the EU Common Market [3]. However, certification (CE marking) of SARS-CoV-2 diagnostics is currently done solely by the manufacturer (self-certification), without third party intervention. The exception are SARS-CoV-2 self-tests, where a notified body has to assess studies with lay persons performing the tests. However, owing to the urgency in the coronavirus disease (COVID-19) situation, a national derogation for CE-certification of self-tests can be agreed by the national competent authority, e. g. by relying on the performance of the same RDT cassette offered for professional use. Starting from May 2022, the IVDD will be replaced by the IVD Regulation (EU) 2017/746 (IVDR) where a risk-based classification of IVD is the basis for the scrutiny of their assessment [4,5]. The SARS-CoV-2 IVD will belong to the high-risk devices (class D) under the IVDR, requiring a notified body both for certification of the manufacturer`s quality management system and for assessment of the technical documentation of the device. Furthermore, EU reference laboratories (EURL) will be responsible for independent laboratory testing of class D devices to verify performance features and to assure batch-to-batch consistency [4]. However, for the time being, independent evaluations of SARS-CoV-2 Ag RDT that allow conclusions on their performance are largely missing.

In the current situation with absence of strict regulatory requirements for most SARS-CoV-2 IVD, the German Ministry of Health decided to link the reimbursement of SARS-CoV-2 Ag RDT to provision of evidence of essential quality features of these assays. This evidence consisted of two parts: (i) compliance with minimum criteria for RDT sensitivity (detection of > 80% of PCR-positive symptomatic patients during the first 7 days after symptom onset) and specificity (> 97% for asymptomatic persons) in studies performed by or on behalf of the manufacturer with clinical specimens, and (ii) successful outcome in the independent laboratory evaluation. Minimum criteria were jointly defined by the Paul-Ehrlich Institute (PEI) and the Robert Koch Institute (RKI), two governmental authorities in Germany [6]. Manufacturers or distributors of SARS-CoV-2 Ag RDT document for the respective SARS-CoV-2 Ag RDT compliance with these criteria before the device can be listed as eligible for reimbursement on a dedicated webpage of Bundesinstitut für Arzneimittel und Medizinprodukte (BfArM), another governmental authority [7].

We selected devices from the BfArM list for the comparative evaluation performed by PEI/RKI. The aim of this comparative evaluation was to both determine the state of the art sensitivity of proficient devices and identify devices not reaching the minimum sensitivity level. The concept of 'state of the art' is also mentioned in the IVD Regulation (EU) 2017/746 (IVDR) [5], describing a defined level of quality features achieved by the majority of assays at a certain time point after their comparative evaluation using an uniform sample set (head-to-head comparison); with continuous improvement of devices, the state of the art level increases over time and would therefore need to be re-assessed at certain intervals. Subsequently, devices with sensitivity below state of the art are removed from the BfArM list while all devices with successful evaluation outcome are published on the PEI webpage [8]. We evaluated 122 SARS-CoV-2 Ag-RDT in direct comparison using a common panel of SARS-CoV-2 specimens. 

## Methods

### Evaluation panel

Detailed characterisation of the evaluation panel has been described by Puyskens et al., published in this issue of *Eurosurveillance* [9]. In short, pools from nasopharyngeal and oropharyngeal swabs from SARS-CoV-2-positive individuals were prepared as random mixtures obtained from up to 10 swabs. While dry swabs were directly eluted in phosphate-buffered saline (PBS), the residual amount of virus transport media (VTM) contained in moist swabs was diluted in PBS. Care was taken not to use VTM containing the protein-denaturing component guanidinium.

Individual pools were composed of samples with similar SARS-CoV-2 concentrations, expressed as quantification cycle (Cq) values of semiquantitative PCR. In total 50 different pools were defined as members of the evaluation panel and stored as 500 µl aliquots at −80 °C. The Cq of each panel member was determined by PCR, and the putative number of RNA copies calculated with the aid of the reference preparations distributed by the German external quality assessment (EQA) provider INSTAND e. V [10]. Furthermore, presence of infectious virus detectable by successful propagation in Vero cell culture was investigated for the individual pools, and results were widely in line with published findings that in vitro infectivity corresponds with virus concentrations of Cq ≤ 25 [11-13]. This finding is widely confirmed in our study with nine of 17 and three of 18 members of the two panel versions 1V1 and 1V2, respectively (see Supplement: Design and manufacture of the evaluation panel). However, there is no established Cq cut–off value at which individuals are estimated to be no longer infectious. 

The whole evaluation panel may be subdivided into three subgroups: panel members, which are characterised by very high (Cq 17–25; 18 pools), high (Cq >25– <30; 23 pools) or moderate (Cq 30–36; nine pools) viral load. During the comparative evaluation, four members of the original panel (1V1) had to be replaced, resulting in a slight shift in the subgroup composition in the resulting panel 1V2: 17 pools covering the Cq range 17–25, 23 pools the Cq range >25– <30 and 10 pools the Cq range 30–36.

### Antigen stability

Real-time antigen stability in panel members was investigated at the PEI using quantitative SARS-CoV-2 ELISA Lumipulse G SARS-CoV-2 Ag (Fujirebio Inc., Shinjuku-ku, Tokyo, Japan). Panel members were tested after initial thawing and 1 week incubation at 4 °C. Furthermore, potential impact of an additional freeze/thaw cycle was addressed.

### Comparative evaluation

In the beginning of the comparative evaluation, participating laboratories included those at the RKI, the PEI, the Nationales Konsiliarlaboratorium für Coronaviren (Institute of Virology, Charité), the Bundeswehr Institute of Microbiology, the Bernhard-Nocht-Institut für Tropenmedizin and laboratories of the association Akkreditierte Labors in der Medizin (ALM). At a later stage, because of the increasing work load, the evaluation was continued by PEI and RKI. Panels were shipped on dry ice and, once thawed, 50 µL aliquots were prepared, kept at 4 °C and used within 5 days, without further freeze/thaw step. For each Ag RDT and panel member, the 50 µL aliquot was completely absorbed using the specimen collection device, e.g. a swab, provided with the respective test. The swabs were then eluted in the test-specific buffer, strictly following the respective instructions for use (IFU). After applying the sample/buffer solution onto the test cassette and incubating, visual read-out of control and target lines was done independently by two laboratory technicians, with potential discrepant results preliminarily interpreted as equivocal. Appearance of the RDT control line is a precondition for any valid test result. In favour of the tests evaluated, both reactive and equivocal results for the target line of the RDT were eventually scored as positive. At the PEI, the test cassettes were immediately scanned using BLOTrix Reader R2L (BioSciTec GmbH) and analysed with BLOTrix 4 Cubos (B4C) software (BioSciTec GmbH); at other evaluation sites, the test results were documented as photographs. Some tests were provided with reading instruments and read as per instruction manual provided.

Tests were selected from original manufacturers, as far as this information was available. Often duplicate versions of the very same tests are marketed under a new test name, new manufacturer or different distributor. Repeat testing of duplicates was avoided as far as possible in order to cope with the already large variety of different tests placed on the EU Common Market.

## Results

### Characterisation of the evaluation panel

Panel members spanned the Cq range between 17 and 36. A specimen with an assigned SARS-CoV-2 RNA concentration of 10^6^ RNA copies/mL provided by INSTAND corresponded to the Cq value of 25. Assuming that a Cq difference of 1 corresponds to a concentration factor of 2, and taking into account that the individual panel members covered a Cq range from 17 to 36, the SARS-CoV-2 RNA amounts in the panel covered a concentration range from > 10^8^ to < 10^3^ copies per mL, respectively. The Cq values of 20 or 30 therefore corresponded to approximate SARS-CoV-2 RNA concentrations of 3 × 10^7^ or 3 × 10^4^ copies per mL, respectively. SARS-CoV-2 propagation in cell culture resulted in positive results for several of the low Cq/high-titre specimens, indicating presence of infectious virus despite the various preparation steps (more details in [9] and in the Supplement: Design and manufacture of the evaluation panel).

Investigation of stability of the analyte SARS-CoV-2 antigen in panel members revealed a negative effect for additional freeze/thaw steps; in contrast, there was no obvious impact on the antigen content after 7 days experimental storage at 4 °C of the liquid 50 µL aliquots (data not shown). From one 500 µL thawed aliquot, routinely nine to 10 aliquots of 50 µL were immediately filled and used within 5 days for evaluation of nine to 10 RDT, respectively, ensuring that there were no stability issues.

### Comparative evaluation

We evaluated 122 SARS-CoV-2 rapid tests in direct comparison using the evaluation panel, with only minor differences in composition between the closely related panel versions 1V1 and 1V2 (see Supplementary Figures S1 and S2). For acceptable Ag RDT performance, we defined a minimum detection rate (sensitivity) of 75% for the panel member subgroup with very high SARS-CoV-2 concentration (Cq ≤ 25, viral load around 10^6^ SARS-CoV-2 RNA/mL and higher). This criterion corresponds to the detection of at least 14 of 18 positives in this subgroup of panel 1V1 (18 members with Cq ≤ 25), or 13 of 17 in panel 1V2. 

Of the 122 SARS-CoV-2 Ag RDT evaluated, 96 tests (79%) had a sensitivity of > 75% for panel members with high viral loads (Cq ≤ 25; Table 1), and 26 tests (21%) were of lower sensitivity not meeting the sensitivity criterion (Table 2). Of the 96 tests meeting the sensitivity limit, 58 (60%) detected all panel members of the subgroup with Cq ≤ 25 (100% subgroup sensitivity), and another 17 tests (18%) exhibited a respective subgroup sensitivity of > 90%. In addition, 20 tests (20.8%) showed a detection rate of > 75% even in the Cq range >25– <30. Table 3 lists the subgroups based on performance data.

**Table 1:** Comparative evaluation results of SARS-CoV-2 antigen rapid diagnostic tests passing the sensitivity criteria (in alphabetical order of manufacturers), Germany, 2020–2021 (n = 96)

RDT	Manufacturer	Test name	Sensitivity			
			Cq ≤ 25	Cq >25– <30	Cq ≥ 30	Cq 17–36
1	Abbott Rapid Diagnostics Jena GmbH	PanbioCOVID-19 Ag Rapid Test Device (NASOPHARYNGEAL)	100.0%	60.9%	0.0%	64.0%
2	ACON Biotech (Hangzhou) Co., Ltd	Flowflex SARS-CoV-2-Antigenschnelltest (Nasopharynxtupfer)	94.1%	4.3%	0.0%	34.0%
3	Aesku Diagnostics GmbH	Aesku Rapid SARS-CoV-2 Rapid Test	82.4%	17.4%	0.0%	36.0%
4	Affimedix	TestNOW - COVID-19 Antigen	100.0%	47.8%	0.0%	58.0%
5	Amazing Biotech (Shanghai) Co., Ltd	CoroVisio Covid-19 Ag Versieglungsröhrchen Teststreifen (Kolloidales Gold)	76.5%	8.7%	0.0%	30.0%
6	Ameda Labordiagnostik GmbH	AMP Rapid Test SARS-CoV-2 Ag	100.0%	78.3%	0.0%	70.0%
7	AmonMed (Xiamen) Biotechnology Co., Ltd.	COVID-19 Antigen Rapid Test Kit (Colloidal Gold)	100.0%	87.0%	30.0%	80.0%
8	Anbio (Xiamen) Biotechnology Co., Ltd	Rapid Covid-19 Antigen Test (Colloidal Gold)	100.0%	52.2%	0.0%	58.0%
9	Anhui Deepblue Medical Technology Co., Ltd.	COVID-19 (SARS CoV-2) Antigen Test Kit (Colloidal Gold)	100.0%	39.1%	0.0%	52.0%
10	ASAN PHARM.CO., LTD.	Asan Easy Test COVID-19 Ag	100.0%	69.6%	0.0%	66.0%
11	Atlas Link Technology Co., Ltd.	Nova Test SARS-CoV-2 Antigen Rapid Test Kit	100.0%	60.9%	0.0%	62.0%
12	Avalun	Ksmart SARS-COV2 Antigen Rapid Test	94.1%	13.0%	0.0%	38.0%
13	AXIOM Gesellschaft für Diagnostica und Biochemica mbH	Axiom Diagnostics COVID-19 Ag Schnelltest	100.0%	52.2%	0.0%	58.0%
14	Azure Biotech Inc.	Dia Sure Covid-19 Antigen Rapid Test Device (Nasopharyngeal/oropharyngeal swab)	76.5%	13.0%	20.0%	36.0%
15	Becton Dickinson	BD Veritor System for Rapid Detection of SARS-CoV-2	83.3%	8.7%	0.0%	34.0%
16	Beijing Beier Bioengineering Co., Ltd.	Covid-19 Antigen Schnelltest	77.8%	0.0%	0.0%	28.0%
17	Beijing Hotgen Biotech Co., Ltd.	Novel Coronavirus 2019-nCoV Antigen Test (Colloidal gold)	100.0%	47.8%	0.0%	56.0%
18	Beijing Lepu Medical Technology Co., Ltd	SARS-CoV-2 Antigen Rapid Test Kit	100.0%	26.1%	0.0%	46.0%
19	Beijing Tigsun Diagnostics Co.;Ltd.	Tigsun COVID-19 Saliva Antigen Rapid Test	100.0%	87.0%	30.0%	80.0%
20	BIOMERICA Inc.	COVID-19-Antigen-Schnelltest (Nasopharyngeal-Abstrich)	100.0%	30.4%	0.0%	48.0%
21	BIONOTE	NowCheck COVID-19 Ag Test	100.0%	65.2%	0.0%	66.0%
22	BioRepair GmbH	Covid 19 Antigen Schnelltest	100.0%	78.3%	0.0%	70.0%
23	BIOSYNEX SWISS SA	BIOSYNEX COVID-19 Ag BSS	100.0%	78.3%	11.1%	74.0%
24	BTNX, Inc. (Biotrend Chemikalien GmbH)	Rapid Response COVID-19 Rapid Test Device	94.1%	13.0%	10.0%	40.0%
25	Chil Tibbi Mal. San. Tic. Ltd. Şti	COVID-19 Antigen Schnell Test (Nasopharyngeal / Oropharyngeal Tupfer Kassette)	100.0%	60.9%	0.0%	62.0%
26	Core Technology Co., Ltd.	Canea COVID-19 Antigen Schnelltest	88.2%	26.1%	0.0%	42.0%
27	DNA Diagnostic A/S.	Covid-19 Antigen Detection Kit	100.0%	39.1%	10.0%	54.0%
28	Edinburgh Genetics Limited	Edinburgh Genetics ActivXpress + COVID-19 Antigen Complete Testing Kit	100.0%	34.8%	0.0%	50.0%
29	Eurobio Scientific	EBS SARS-CoV-2 Ag Rapid Test	94.1%	34.8%	0.0%	48.0%
30	Fujirebio Inc. (Mast Diagnostica GmbH)	ESPLINE SARS-CoV-2	100.0%	21.7%	0.0%	46.0%
31	Genrui Biotech Inc.	Genrui SARS-CoV-2 Antigen Test Kit (Colloidal Gold)	94.1%	56.5%	0.0%	58.0%
32	GenSure Biotech Inc.	DIA-COVID COVID-19 Ag Rapid Test Kit	94.1%	13.0%	0.0%	38.0%
33	Getein Biotech, Inc.	One Step Test for SARS-CoV-2 Antigen (Colloidal Gold)	100.0%	82.6%	0.0%	72.0%
34	Green Cross Medical Science Corp. (Weko Pharma GmbH)	Genedia W Covid-19 Ag	83.3%	8.7%	0.0%	34.0%
35	Guangdong Hecin Scientific,Inc.	2019-nCoV Antigen Test Kit(colloidal gold method)	82.4%	13.0%	0.0%	34.0%
36	Guangdong Wesail Biotech Co., Ltd.	COVID-19 Ag Test Kit	100.0%	52.2%	11.1%	62.0%
37	Guangzhou Wondfo Biotech Co. Ltd	Wondfo SARS-CoV-2 Antigen Test (Lateral Flow Method)	88.2%	0.0%	0.0%	30.0%
38	Hangzhou Clongene Biotech Co., Ltd.	Clungene COVID-19 Antigen Rapid Test	94.4%	34.8%	0.0%	50.0%
39	Hangzhou Immuno Biotech Co.,Ltd.	IMMUNOBIO SARS-CoV-2 Antigen-Schnelltest (COVID-19 Ag)	88.2%	13.0%	0.0%	36.0%
40	Hangzhou Laihe Biotech Co., Ltd. (Lissner Qi GmbH)	Lyher Novel Coronavirus (COVID-19) Antigen Test Kit (Colloidal Gold)	94.4%	17.4%	0.0%	42.0%
41	Hangzhou Lysun Biotechnology Co., Ltd.	Lysun COVID-19 Antigen Rapid Test Device (Colloidal Gold)	100.0%	78.3%	0.0%	70.0%
42	Hangzhou Testsea Biotechnology Co., Ltd	Testsealabs Rapid Test Kit COVID-19 Antigen Test Cassette	100.0%	47.8%	0.0%	56.0%
43	Humasis Co., Ltd.	Humasis COVID-19 Ag Test	88.2%	21.7%	0.0%	40.0%
44	IVC Pragen Healthcare	GenBody COVID-19 Ag	94.4%	26.1%	0.0%	46.0%
45	Jiangsu Diagnostics Biotechnology Co., Ltd	COVID-19 Antigen Rapid Test Cassette (Colloidal Gold)	100.0%	78.3%	0.0%	68.0%
46	Jiangsu Medomics Medical Technology Co., Ltd	SARS-CoV-2-Antigen-Testkit (LFIA)	94.1%	21.7%	0.0%	42.0%
47	Joinstar Biomedical Technology Co., Ltd (CIV care impuls Vertrieb)	COVID-19 Antigen Schnelltest (Colloidal Gold)	100.0%	60.9%	0.0%	64.0%
48	Labnovation Technologies, Inc.	Labnovation SARS-CoV-2 Antigen Rapid Test Kit (Immunochromatography)	94.1%	17.4%	0.0%	40.0%
49	Lumigenex (Suzhou) Co., Ltd.	PocRoc SARS-CoV-2, Antigen Schnelltest Set (Kolloidales Gold)	100.0%	65.2%	0.0%	64.0%
50	LumiQuick Diagnostics, Inc.	QuickProfile Covid-19 Antigen Test Card	100.0%	91.3%	20.0%	80.0%
51	LumiraDX	LumiraDx SARS-CoV-2 Ag Test	100.0%	52.2%	0.0%	60.0%
52	MEDsan GmbH	MEDsan SARS-CoV-2 Antigen Rapid Test	100.0%	47.8%	0.0%	58.0%
53	Merlin Biomedical (Xiamen) Co., Ltd.	SARS-CoV-2 Antigen Rapid Test Cassette	100.0%	82.6%	0.0%	72.0%
54	Mölaboratory GmbH	mö-screen Corona Antigen Test	100.0%	47.8%	0.0%	58.0%
55	MP Biomedicals Germany GmbH	Rapid SARS-CoV-2 Antigen Test Card	100.0%	43.5%	0.0%	54.0%
56	nal von minden gmbh	NADAL COVID-19 Ag Schnelltest	83.3%	13.0%	0.0%	36.0%
57	Nanjing Norman Biological Technology Co.,Ltd	Novel Coronavirus (2019-nCOV) Antigen Testing Kit (Colloidal Gold)	94.1%	26.1%	0.0%	44.0%
58	NanoEntek Inc	FRENDTM COVID-19 Ag	88.2%	8.7%	0.0%	34.0%
59	Nantong Diagnos Biotechnology Co., Ltd.	COVID-19 Antigen Saliva Test Kit (Colloidal Gold)	100.0%	56.5%	0.0%	60.0%
60	New Gene (Hangzhou) Bioengineering Co., Ltd.	Covid-19-Antigen-Testkit	100.0%	87.0%	20.0%	78.0%
61	Novatech Tibbi Cihaz Ürünleri San. Ve Tic.A.S.	novacheck-Ag SARS-CoV-2 Covid-19 Antigen Rapid Test	94.1%	21.7%	0.0%	42.0%
62	Oncosem Onkolojik Sistemler San. Ve Tic. A.S.	CAT Antigen Covid Rapid Test	94.1%	30.4%	0.0%	46.0%
63	PCL, Inc.	PCL COVID19 Ag Gold Saliva	100.0%	52.2%	0.0%	58.0%
64	PerGrande BioTech Development Co., Ltd.	SARS-CoV-2 Antigen Detection Kit (Colloidal Gold Immunochromatographic Assay)	100.0%	17.4%	0.0%	42.0%
65	Precision Biosensor Inc. (Axon Laboratory AG)	Exdia COVID-19-Ag-Test	100.0%	60.9%	0.0%	64.0%
66	ProGnosis Biotech	Rapid Test Ag 2019-nCoV	94.1%	65.2%	10.0%	64.0%
67	Quidel Corporation	Sofia SARS Antigen FIA	88.9%	8.7%	0.0%	36.0%
68	Qingdao Hightop Biotech Co., Ltd.	Hightop SARS-CoV-2 (Covid-19) Antigen Rapid Test	100.0%	43.5%	0.0%	54.0%
69	R-Biopharm AG	RIDAQUICK SARS-CoV-2 Antigen	100.0%	17.4%	0.0%	44.0%
70	Safecare Biotech Hangzhou Co., Ltd.	Safecare COVID-19 Ag Rapid Test Kit (Swab)	100.0%	60.9%	0.0%	62.0%
71	Salofa OY	salocor SARS-CoV-2 Antigen Rapid Test Cassette (Nasopharyngeal swab)	82.4%	13.0%	0.0%	34.0%
72	ScheBo Biotech AG	ScheBo SARS-CoV-2 Quick Antigen	100.0%	91.3%	10.0%	78.0%
73	SD BIOSENSOR (Roche Diagnostics GmbH)	SARS-CoV-2 Rapid Antigen Test	88.9%	30.4%	0.0%	46.0%
74	SD BIOSENSOR	STANDARD Q COVID-19 Ag Test	88.9%	30.4%	0.0%	46.0%
75	SD BIOSENSOR	STANDARD F COVID-19 Ag FIA	100.0%	65.2%	0.0%	66.0%
76	SGA Mühendislik DAN. EG. Icve DIS.Ltd.STI	V-Chek SARS-CoV-2 Rapid Ag Test Kit (Colloidal Gold)	94.1%	26.1%	0.0%	44.0%
77	Shenzhen Lvshiyuan Biotechnology Co., Ltd.	Green Spring SARS-CoV-2 Antigen Rapid Test Kit (Colloidal Gold)	100.0%	95.7%	40.0%	86.0%
78	Shenzhen Microprofit Biotech Co., Ltd	fluorecare COVID-19 SARS-CoV-2 Spike Protein Test Kit (Colloidal Gold Chromatographic Immunoassay)	100.0%	47.8%	10.0%	58.0%
79	Shenzhen Watmind Medical Co.,Ltd.	SARS-CoV-2 Ag Diagnostic Test Kit (Colloidal Gold)	100.0%	95.7%	20.0%	82.0%
80	Shenzhen Watmind Medical Co.,Ltd.	SARS-CoV-2 Ag Diagnostic Test Kit (Immuno-fluorescence)	100,0%	60.9%	0.0%	62.0%
81	Shenzhen Zhenrui Biotech co. Ltd.	Zhenrui COVID-19 (SARS-COV-2) Antigen Test Kits	82.4%	13.0%	0.0%	34.0%
82	Siemens Healthineers	CLINITEST Rapid COVID-19 Antigen Test	100.0%	87.0%	0.0%	76.0%
83	Sugentech, Inc.	SGTi-flex COVID-19 Ag	100.0%	73.9%	0.0%	68.0%
84	Toda Pharma	Toda Coronadiag Ag	100.0%	95.7%	40.0%	86.0%
85	Triplex International Biosciences (China) Co., Ltd.	SARS-CoV-2 Antigen Rapid Test Kit	100.0%	87.0%	20.0%	78.0%
86	ulti med Products (Deutschland) GmbH	COVID-19 Antigen Speicheltest (Immunochromatographie)	100.0%	95.7%	20.0%	82.0%
87	Vitrosens Biyoteknoloji Ltd. Sti	RapidFor SARS-CoV-2 Rapid Antigen Test Colloidal Gold	100.0%	30.4%	0.0%	48.0%
88	Wantai (Beijing Wantai Biological Pharmacy Enterprise Co., Ltd.)	SARS-CoV-2 Ag Rapid Test (FIA)	100.0%	78.3%	0.0%	72.0%
89	Wuhan EasyDiagnosis Biomedicine Co., Ltd	COVID-19 (SARS-CoV-2) Antigen Test Kit	100.0%	73.9%	0.0%	68.0%
90	Wuhan Life Origin Biotech Joint Stock Co., Ltd.	SARS-CoV-2 Antigen Assay Kit (Immunochromatography)	100.0%	56.5%	0.0%	60.0%
91	Wuhan UNscience Biotechnology Co., Ltd.	SARS-CoV-2 Antigen Rapid Test Kit	88.2%	17.4%	0.0%	38.0%
92	Xiamen Boson Biotech Co., Ltd	SARS-CoV-2 Antigen Schnelltest	100.0%	43.5%	0.0%	54.0%
93	Xiamen WIZ Biotech Co., Ltd.	Wizbiotech SARS-CoV-2 Antigen Rapid Test	88.2%	13.0%	0.0%	36.0%
94	Zet Medikal Tekstil Dis Ticaret Ltd. STI.	softec SARS COV-2 (Covid-19) Antigen Test Kit	82.4%	21.7%	10.0%	40.0%
95	Zhejiang Anji Saianfu Biotech Co., Ltd.	reOpenTest COVID-19 Antigen Rapid Test (Colloidal Gold)	94.1%	30.4%	0.0%	46.0%
96	Zhejiang Orient Gene Biotech Co., Ltd	Coronavirus Ag Rapid Test Cassette (Swab)	100.0%	87.0%	0.0%	76.0%

Cq: quantitative cycle; SARS-CoV-2: severe acute respiratory syndrome coronavirus 2.

Criteria as defined by detection rate of >75% in panel subgroup with Cq ≤ 25.

**Table 2:** Comparative evaluation results of SARS-CoV-2 antigen rapid diagnostic tests missing the sensitivity criteria (in alphabetical order of manufacturers), Germany, 2020–2021 (n = 26)

RDT	Manufacturer	Test name	Sensitivity			
			Cq ≤ 25	Cq >25– <30	Cq ≥ 30	Cq 17–36
97	Acro Biotech Inc	Acro COVID-19 Antigen Rapid Test	16.7%	0.0%	0.0%	6.0%
98	Aikang Diagnostics Co., Ltd.	SARS-CoV-2 Antigen Test Kit (Immunochromatography)	11.8%	0.0%	0.0%	4.0%
99	Beijing Savant Biotechnology Co., Ltd	New Coronavirus (SARS-CoV-2) N Protein Detection Kit (Fluorescence Immunchromatography)	0.0%	0.0%	0.0%	0.0%
100	CertestT Biotec S. L.	CerTest Biotec SARS-CoV-2 Ag Test	29.4%	0.0%	0.0%	10.0%
101	Coris Bioconcept	COVID-19 Ag Respi-Strip	33.3%	0.0%	0.0%	12.0%
102	Hangzhou AllTest Biotech Co. Ltd.	COVID-19 AG AllTest	16.7%	0.0%	0.0%	6.0%
103	Hangzhou Biotest Biotech Co., Ltd.	Lumiratek SARS-CoV-2 Antigen Rapid Test Cassette	29.4%	0.0%	0.0%	10.0%
104	Hangzhou Genesis Biocontrol Co., Ltd	KaiBiLi COVID-19 Antigen Rapid Test Device	52.9%	0.0%	0.0%	18.0%
105	Hangzhou Realy Tech Co., Ltd.	Novel Coronavirus (SARS-Cov-2) Antigen Rapid Test Cassette (swab)	58.8%	0.0%	0.0%	20.0%
106	Inzek International Trading	Biozek medical COVID-19 Antigen Rapid Test Cassette	52.9%	0.0%	0.0%	18.0%
107	Joinstar Biomedical Technology Co., Ltd	COVID-19 Antigen Rapid Test (Latex)	0.0%	0.0%	0.0%	0.0%
108	Joysbio (Tianjin) Biotechnology Co., Ltd.	Joysbio SARS-CoV-2 Antigen Rapid Test Kit (Colloidal Gold)	47.1%	4.3%	0.0%	18.0%
109	Lionex GmbH	Lionex COVID-19 Ag Rapid Test	0.0%	0.0%	0.0%	0.0%
110	Medicon Co., Ltd.	Trueline COVID-19 Ag Rapid Test	58.8%	4.3%	0.0%	22.0%
111	Mexacare GmbH Heidelberg	QuickTestCorona COVID-19 Antigen Schnelltest	52.9%	4.3%	0.0%	20.0%
112	nal von minden GmbH	dedicio Medical Test COVID-19 Ag plus Test	35.3%	0.0%	0.0%	12.0%
113	Rapigen	Biocredit COVID-19 Ag	16.7%	0.0%	0.0%	6.0%
114	Servoprax	Cleartest Coronaantigen	66.7%	0.0%	0.0%	24.0%
115	Spring Healthcare Services SP zoo	SARS-Cov-2 Antigen Rapid Test Cassette (swab)	29.4%	0.0%	0.0%	10.0%
116	SureScreen Diagnostics Ltd	COVID-19 Antigen Rapid Test Cassette	52.9%	0.0%	0.0%	18.0%
117	TaiDoc Technology Corp.	FORA COVID-19 ANTIGEN RAPID TEST	27.8%	0.0%	0.0%	10.0%
118	Unioninvest	Unibioscience COVID-19 Rapid Antigen Test	0.0%	0.0%	0.0%	0.0%
119	VivaChek Biotech (Hangzhou) Co, Ltd.	VivaDiag SARS-CoV-2 Ag Rapid Test	50.0%	0.0%	0.0%	18.0%
120	VivaChek Biotech (Hangzhou) Co, Ltd.	VivaDiag Pro SARS-CoV-2 Ag Rapid Test	64.7%	0.0%	0.0%	22.0%
121	W.H.P.M, Inc	First SIGN SARS-CoV-2 Antigen Test	47.1%	0.0%	0.0%	16.0%
122	Xiamen Zhongsheng Langjie Biotechnology Co., Ltd	Covid-19 Antigen Test Cassette	11.8%	0.0%	0.0%	4.0%

Cq: quantitative cycle; SARS-CoV-2: severe acute respiratory syndrome coronavirus 2.

Criteria as defined by detection rate of >75% in panel subgroup with Cq ≤ 25.

**Table 3:** Comparative evaluation results of SARS-CoV-2 antigen rapid diagnostic tests passing the sensitivity criteria (sorted by performance in the subgroups of the evaluation panel), Germany, 2020–2021 (n = 96)

RDT	Manufacturer	Test name	Sensitivity			
			Cq ≤ 25	Cq >25– <30	Cq ≥ 30	Cq 17–36
Subgroup of RDT with detection rates of 100% for Cq ≤ 25 and of >75% for Cq >25– <30						
77	Shenzhen Lvshiyuan Biotechnology Co., Ltd.	Green Spring SARS-CoV-2 Antigen Rapid Test Kit (Colloidal Gold)	100.0%	95.7%	40.0%	86.0%
84	Toda Pharma	Toda Coronadiag Ag	100.0%	95.7%	40.0%	86.0%
79	Shenzhen Watmind Medical Co.,Ltd.	SARS-CoV-2 Ag Diagnostic Test Kit (Colloidal Gold)	100.0%	95.7%	20.0%	82.0%
86	ulti med Products (Deutschland) GmbH	COVID-19 Antigen Speicheltest (Immunochromatographie)	100.0%	95.7%	20.0%	82.0%
50	LumiQuick Diagnostics, Inc.	QuickProfile Covid-19 Antigen Test Card	100.0%	91.3%	20.0%	80.0%
72	ScheBo Biotech AG	ScheBo SARS-CoV-2 Quick Antigen	100.0%	91.3%	10.0%	78.0%
7	AmonMed (Xiamen) Biotechnology Co., Ltd.	COVID-19 Antigen Rapid Test Kit (Colloidal Gold)	100.0%	87.0%	30.0%	80.0%
19	Beijing Tigsun Diagnostics Co.;Ltd.	Tigsun COVID-19 Saliva Antigen Rapid Test	100.0%	87.0%	30.0%	80.0%
60	New Gene (Hangzhou) Bioengineering Co., Ltd.	Covid-19-Antigen-Testkit	100.0%	87.0%	20.0%	78.0%
85	Triplex International Biosciences (China) Co., Ltd.	SARS-CoV-2 Antigen Rapid Test Kit	100.0%	87.0%	20.0%	78.0%
96	Zhejiang Orient Gene Biotech Co., Ltd	Coronavirus Ag Rapid Test Cassette (Swab)	100.0%	87.0%	0.0%	76.0%
82	Siemens Healthineers	CLINITEST Rapid COVID-19 Antigen Test	100.0%	87.0%	0.0%	76.0%
33	Getein Biotech, Inc.	One Step Test for SARS-CoV-2 Antigen (Colloidal Gold)	100.0%	82.6%	0.0%	72.0%
53	Merlin Biomedical (Xiamen) Co., Ltd.	SARS-CoV-2 Antigen Rapid Test Cassette	100.0%	82.6%	0.0%	72.0%
22	BioRepair GmbH	Covid 19 Antigen Schnelltest	100.0%	78.3%	0.0%	70.0%
6	Ameda Labordiagnostik GmbH	AMP Rapid Test SARS-CoV-2 Ag	100.0%	78.3%	0.0%	70.0%
23	BIOSYNEX SWISS SA	BIOSYNEX COVID-19 Ag BSS	100.0%	78.3%	11.1%	74.0%
41	Hangzhou Lysun Biotechnology Co., Ltd.	Lysun COVID-19 Antigen Rapid Test Device (Colloidal Gold)	100.0%	78.3%	0.0%	70.0%
45	Jiangsu Diagnostics Biotechnology Co., Ltd	COVID-19 Antigen Rapid Test Cassette (Colloidal Gold)	100.0%	78.3%	0.0%	68.0%
88	Wantai (Beijing Wantai Biological Pharmacy Enterprise Co., Ltd.)	SARS-CoV-2 Ag Rapid Test (FIA)	100.0%	78.3%	0.0%	72.0%
Subgroup of RDT with detection rates of 100% for Cq ≤ 25 and of <75% for Cq >25– <30						
83	Sugentech, Inc.	SGTi-flex COVID-19 Ag	100.0%	73.9%	0.0%	68.0%
89	Wuhan EasyDiagnosis Biomedicine Co., Ltd	COVID-19 (SARS-CoV-2) Antigen Test Kit	100.0%	73.9%	0.0%	68.0%
10	ASAN PHARM.CO.,LTD.	Asan Easy Test COVID-19 Ag	100.0%	69.6%	0.0%	66.0%
49	Lumigenex (Suzhou) Co., Ltd.	PocRoc SARS-CoV-2, Antigen Schnelltest Set (Kolloidales Gold)	100.0%	65.2%	0.0%	64.0%
75	SD BIOSENSOR	STANDARD F COVID-19 Ag FIA	100.0%	65.2%	0.0%	66.0%
21	BIONOTE	NowCheck COVID-19 Ag Test	100.0%	65.2%	0.0%	66.0%
1	Abbott Rapid Diagnostics Jena GmbH	PanbioCOVID-19 Ag Rapid Test Device (NASOPHARYNGEAL)	100.0%	60.9%	0.0%	64.0%
11	Atlas Link Technology Co., Ltd.	Nova Test SARS-CoV-2 Antigen Rapid Test Kit	100.0%	60.9%	0.0%	62.0%
25	Chil Tibbi Mal. San. Tic. Ltd. Şti	COVID-19 Antigen Schnell Test (Nasopharyngeal / Oropharyngeal Tupfer Kassette)	100.0%	60.9%	0.0%	62.0%
47	Joinstar Biomedical Technology Co., Ltd (CIV care impuls Vertrieb)	COVID-19 Antigen Schnelltest (Colloidal Gold)	100.0%	60.9%	0.0%	64.0%
65	Precision Biosensor Inc. (Axon Laboratory AG)	Exdia COVID-19-Ag-Test	100.0%	60.9%	0.0%	64.0%
70	Safecare Biotech Hangzhou Co., Ltd.	Safecare COVID-19 Ag Rapid Test Kit (Swab)	100.0%	60.9%	0.0%	62.0%
80	Shenzhen Watmind Medical Co.,Ltd.	SARS-CoV-2 Ag Diagnostic Test Kit (Immuno-fluorescence)	100.0%	60.9%	0.0%	62.0%
59	Nantong Diagnos Biotechnology Co., Ltd.	COVID-19 Antigen Saliva Test Kit (Colloidal Gold)	100.0%	56.5%	0.0%	60.0%
90	Wuhan Life Origin Biotech Joint Stock Co., Ltd.	SARS-CoV-2 Antigen Assay Kit (Immunochromatography)	100.0%	56.5%	0.0%	60.0%
36	Guangdong Wesail Biotech Co., Ltd.	COVID-19 Ag Test Kit	100.0%	52.2%	11.1%	62.0%
8	Anbio (Xiamen) Biotechnology Co., Ltd	Rapid Covid-19 Antigen Test (Colloidal Gold)	100.0%	52.2%	0.0%	58.0%
13	AXIOM Gesellschaft für Diagnostica und Biochemica mbH	Axiom Diagnostics COVID-19 Ag Schnelltest	100.0%	52.2%	0.0%	58.0%
51	LumiraDX	LumiraDx SARS-CoV-2 Ag Test	100.0%	52.2%	0.0%	60.0%
63	PCL, Inc.	PCL COVID19 Ag Gold Saliva	100.0%	52.2%	0.0%	58.0%
78	Shenzhen Microprofit Biotech Co., Ltd	fluorecare COVID-19 SARS-CoV-2 Spike Protein Test Kit (Colloidal Gold Chromatographic Immunoassay)	100.0%	47.8%	10.0%	58.0%
17	Beijing Hotgen Biotech Co., Ltd.	Novel Coronavirus 2019-nCoV Antigen Test (Colloidal gold)	100.0%	47.8%	0.0%	56.0%
42	Hangzhou Testsea Biotechnology Co., Ltd	Testsealabs Rapid Test Kit COVID-19 Antigen Test Cassette	100.0%	47.8%	0.0%	56.0%
52	MEDsan GmbH	MEDsan SARS-CoV-2 Antigen Rapid Test	100.0%	47.8%	0.0%	58.0%
54	Mölaboratory GmbH	mö-screen Corona Antigen Test	100.0%	47.8%	0.0%	58.0%
4	Affimedix	TestNOW - COVID-19 Antigen	100.0%	47.8%	0.0%	58.0%
55	MP Biomedicals Germany GmbH	Rapid SARS-CoV-2 Antigen Test Card	100.0%	43.5%	0.0%	54.0%
68	Qingdao Hightop Biotech Co., Ltd.	Hightop SARS-CoV-2 (Covid-19) Antigen Rapid Test	100.0%	43.5%	0.0%	54.0%
92	Xiamen Boson Biotech Co., Ltd	SARS-CoV-2 Antigen Schnelltest	100.0%	43.5%	0.0%	54.0%
27	DNA Diagnostic A/S.	Covid-19 Antigen Detection Kit	100.0%	39.1%	10.0%	54.0%
9	Anhui Deepblue Medical Technology Co., Ltd.	COVID-19 (SARS CoV-2) Antigen Test Kit (Colloidal Gold)	100.0%	39.1%	0.0%	52.0%
28	Edinburgh Genetics Limited	Edinburgh Genetics ActivXpress + COVID-19 Antigen Complete Testing Kit	100.0%	34.8%	0.0%	50.0%
20	BIOMERICA Inc.	COVID-19-Antigen-Schnelltest (Nasopharyngeal-Abstrich)	100.0%	30.4%	0.0%	48.0%
87	Vitrosens Biyoteknoloji Ltd. Sti	RapidFor SARS-CoV-2 Rapid Antigen Test Colloidal Gold	100.0%	30.4%	0.0%	48.0%
18	Beijing Lepu Medical Technology Co., Ltd	SARS-CoV-2 Antigen Rapid Test Kit	100.0%	26.1%	0.0%	46.0%
30	Fujirebio Inc. (Mast Diagnostica GmbH)	ESPLINE SARS-CoV-2	100.0%	21.7%	0.0%	46.0%
69	R-Biopharm AG	RIDAQUICK SARS-CoV-2 Antigen	100.0%	17.4%	0.0%	44.0%
64	PerGrande BioTech Development Co., Ltd.	SARS-CoV-2 Antigen Detection Kit (Colloidal Gold Immunochromatographic Assay)	100.0%	17.4%	0.0%	42.0%
Subgroup of RDT with detection rates of <100% for Cq ≤ 25						
66	ProGnosis Biotech	Rapid Test Ag 2019-nCoV	94.1%	65.2%	10.0%	64.0%
31	Genrui Biotech Inc.	Genrui SARS-CoV-2 Antigen Test Kit (Colloidal Gold)	94.1%	56.5%	0.0%	58.0%
38	Hangzhou Clongene Biotech Co., Ltd.	Clungene COVID-19 Antigen Rapid Test	94.4%	34.8%	0.0%	50.0%
29	Eurobio Scientific	EBS SARS-CoV-2 Ag Rapid Test	94.1%	34.8%	0.0%	48.0%
62	Oncosem Onkolojik Sistemler San. Ve Tic. A.S.	CAT Antigen Covid Rapid Test	94.1%	30.4%	0.0%	46.0%
95	Zhejiang Anji Saianfu Biotech Co., Ltd.	reOpenTest COVID-19 Antigen Rapid Test (Colloidal Gold)	94.1%	30.4%	0.0%	46.0%
44	IVC Pragen Healthcare	GenBody COVID-19 Ag	94.4%	26.1%	0.0%	46.0%
57	Nanjing Norman Biological Technology Co.,Ltd	Novel Coronavirus (2019-nCOV) Antigen Testing Kit (Colloidal Gold)	94.1%	26.1%	0.0%	44.0%
76	SGA Mühendislik DAN. EG. Icve DIS.Ltd.STI	V-Chek SARS-CoV-2 Rapid Ag Test Kit (Colloidal Gold)	94.1%	26.1%	0.0%	44.0%
46	Jiangsu Medomics Medical Technology Co., Ltd	SARS-CoV-2-Antigen-Testkit (LFIA)	94.1%	21.7%	0.0%	42.0%
61	Novatech Tibbi Cihaz Ürünleri San. Ve Tic.A.S.	novacheck-Ag SARS-CoV-2 Covid-19 Antigen Rapid Test	94.1%	21.7%	0.0%	42.0%
40	Hangzhou Laihe Biotech Co., Ltd. (Lissner Qi GmbH)	Lyher Novel Coronavirus (COVID-19) Antigen Test Kit (Colloidal Gold)	94.4%	17.4%	0.0%	42.0%
48	Labnovation Technologies, Inc.	Labnovation SARS-CoV-2 Antigen Rapid Test Kit (Immunochromatography)	94.1%	17.4%	0.0%	40.0%
24	BTNX, Inc. (Biotrend Chemikalien Gmbh)	Rapid Response COVID-19 Rapid Test Device	94.1%	13.0%	10.0%	40.0%
12	Avalun	Ksmart SARS-COV2 Antigen Rapid Test	94.1%	13.0%	0.0%	38.0%
32	GenSure Biotech Inc.	DIA-COVID COVID-19 Ag Rapid Test Kit	94.1%	13.0%	0.0%	38.0%
2	ACON Biotech (Hangzhou) Co., Ltd	Flowflex SARS-CoV-2-Antigenschnelltest (Nasopharynxtupfer)	94.1%	4.3%	0.0%	34.0%
Subgroup of RDT with detection rates of <90% for Cq ≤ 25						
73	SD BIOSENSOR (Roche Diagnostics GmbH)	SARS-CoV-2 Rapid Antigen Test	88.9%	30.4%	0.0%	46.0%
74	SD BIOSENSOR	STANDARD Q COVID-19 Ag Test	88.9%	30.4%	0.0%	46.0%
67	Quidel Corporation	Sofia SARS Antigen FIA	88.9%	8.7%	0.0%	36.0%
26	Core Technology Co., Ltd.	Canea COVID-19 Antigen Schnelltest	88.2%	26.1%	0.0%	42.0%
43	Humasis Co., Ltd.	Humasis COVID-19 Ag Test	88.2%	21.7%	0.0%	40.0%
91	Wuhan UNscience Biotechnology Co., Ltd.	SARS-CoV-2 Antigen Rapid Test Kit	88.2%	17.4%	0.0%	38.0%
39	Hangzhou Immuno Biotech Co., Ltd.	IMMUNOBIO SARS-CoV-2 Antigen-Schnelltest (COVID-19 Ag)	88.2%	13.0%	0.0%	36.0%
93	Xiamen WIZ Biotech Co., Ltd.	Wizbiotech SARS-CoV-2 Antigen Rapid Test	88.2%	13.0%	0.0%	36.0%
58	NanoEntek Inc	FRENDTM COVID-19 Ag	88.2%	8.7%	0.0%	34.0%
37	Guangzhou Wondfo Biotech Co. Ltd	Wondfo SARS-CoV-2 Antigen Test (Lateral Flow Method)	88.2%	0.0%	0.0%	30.0%
56	nal von minden gmbh	NADAL COVID-19 Ag Schnelltest	83.3%	13.0%	0.0%	36.0%
15	Becton Dickinson	BD Veritor System for Rapid Detection of SARS-CoV-2	83.3%	8.7%	0.0%	34.0%
34	Green Cross Medical Science Corp. (Weko Pharma GmbH)	Genedia W Covid-19 Ag	83.3%	8.7%	0.0%	34.0%
94	Zet Medikal Tekstil Dis Ticaret Ltd. STI.	softec SARS COV-2 (Covid-19) Antigen Test Kit	82.4%	21.7%	10.0%	40.0%
3	Aesku Diagnostics GmbH	Aesku Rapid SARS-CoV-2 Rapid Test	82.4%	17.4%	0.0%	36.0%
35	Guangdong Hecin Scientific, Inc.	2019-nCoV Antigen Test Kit(colloidal gold method)	82.4%	13.0%	0.0%	34.0%
71	Salofa OY	salocor SARS-CoV-2 Antigen Rapid Test Cassette (Nasopharyngeal swab)	82.4%	13.0%	0.0%	34.0%
81	Shenzhen Zhenrui Biotech co. Ltd.	Zhenrui COVID-19 (SARS-COV-2) Antigen Test Kits	82.4%	13.0%	0.0%	34.0%
16	Beijing Beier Bioengineering Co., Ltd.	Covid-19 Antigen Schnelltest	77.8%	0.0%	0.0%	28.0%
14	Azure Biotech Inc.	Dia Sure Covid-19 Antigen Rapid Test Device (Nasopharyngeal/Oropharyngeal Swab)	76.5%	13.0%	20.0%	36.0%
5	Amazing Biotech (Shanghai) Co., Ltd	CoroVisio Covid-19 Ag Versieglungsröhrchen Teststreifen (Kolloidales Gold)	76.5%	8.7%	0.0%	30.0%

Cq: quantitative cycle; SARS-CoV-2: severe acute respiratory syndrome coronavirus 2.

Criteria as defined by detection rate of >75% in panel subgroup with Cq ≤ 25.

The 96 tests meeting the sensitivity criteria were reactive with between 14 and 41 members of the 50 members panel (see Supplementary Figure S1). On average throughout all successful tests, 27 panel members (54%) were reactive. Overall reactivity of SARS-CoV-2 Ag RDT strongly followed the analyte concentration throughout the panel, confirming the design of this study (see Supplementary Figures S1 and S2).

The 26 SARS-CoV-2 Ag RDT missing the sensitivity criteria either failed completely (two tests with 0 reactives) or were reactive with two to 12 (average: 6.3) panel members. Again, reactivity was dependent on the analyte concentration throughout the panel members (see Supplementary Figure S2). Two further tests failed because of constant faint background reactivity throughout all panel members; this background reactivity was also seen when using pure extraction buffer and was thus not caused by the panel composition (data not shown). According to information provided by the RDT manufacturers, nucleoprotein is used as target antigen in 112, spike protein in three (Table 1: RDT no.78; Table 2: RDT no. 107 and 109) and both nucleoprotein and spike protein in two assays (Table 1: RDT no. 43 and 64). For five assays, information on the target antigen was not available. Although two of the five assays detecting spike protein failed in this evaluation, the number is too small to conclude on potential association between chosen target antigen and RDT performance.

## Discussion

There is convincing evidence that infectivity of SARS-CoV-2 correlates directly with high viral loads in respiratory specimens of acutely infected persons [11-13]. It has therefore been suggested in many countries to use antigen tests to detect potential infectivity and help control the spread of infection rather than for the purpose of clinical diagnosis. Thus Ag RDT have become a key part of testing strategies since the autumn of 2020. Hundreds of different Ag RDT, most often from East-Asian manufacturers, are available in Europe. Nearly all tests state in their IFU sensitivity values of > 90% for PCR-confirmed specimens. Such statements, being in strong contrast to the results of our study and to other independent evaluations, may be explained by preselection of specimens with strong PCR positivity and/or studies including only few specimens. 

Lack of independent evaluation combined with unjustified statements of quality features led the German Ministry of Health to request in autumn 2020 a comparative evaluation of the sensitivity of test kits offered in Germany. At the time we performed our study (autumn 2020 to spring 2021), there were no EU-wide requirements for quality features of COVID-19 IVD such as a defined minimum sensitivity or minimum specificity, and manufacturers may themselves certify their devices as compliant with basic requirements of the IVDD. Therefore, it was mainly left to individual countries or international organisations to define minimum requirements for the acceptance of tests. However, in autumn 2021, a *Guidance on performance evaluation of SARS-CoV-2 in vitro diagnostic medical devices* was endorsed by the EU Medical Device Coordination Group which will be the basis for future Common Specifications of the IVD Regulation (EU) 2017/746 [14].

In Germany, the Ministry of Health decided to link the reimbursement of SARS-CoV-2 Ag RDT to quality requirements that needed to be fulfilled by acceptable devices. Minimum requirements were jointly formulated by the PEI and RKI and state for SARS-CoV-2 Ag RDT a minimum sensitivity of 80% for PCR-positive specimens obtained within the first 7 days after symptom onset; the minimum specificity was defined as > 97%, and for both requirements, a study population of at least 100 persons is required [6]. Analogous requirements for SARS-CoV-2 Ag RDT have been proposed by the World Health Organization (WHO) for the emergency use listing [15], the United States Food and Drug Administration [16], the European Centre for Disease Prevention and Control [17], the Swiss Authority Bundesamt für Gesundheit [18] and the non-governmental Foundation for Innovative New Diagnostics [19]. Furthermore, RDT reimbursable in Germany had to pass our comparative evaluation, the first part of which is summarised in this manuscript; the evaluation has been continued for further RDT with an equivalent panel version 3 (data not included in this manuscript). 

The definition of 75% minimal detection rate (analytical sensitivity) for panel members with Cq ≤ 25 in our comparative evaluation was based on different reasons. Firstly, infectious virus determined by cell culture propagation was reported for specimens with virus concentrations corresponding to an RNA level of around 10^6^ copies/mL and higher [11-13] (Supplement). Secondly, early in the evaluation, this limit proved to differentiate between RDT with different levels of analytical sensitivity, widely in accordance with diagnostic sensitivity determined in independent SARS-CoV-2 RDT evaluations using clinical specimens [19,20]. Thirdly, there is intrinsic variation between different nucleic acid amplification tests with regard to reported Cq values because of assay-specific nucleic acid extraction/elution volumes combined with assay-specific amplification input volume and amplification efficacy. This fact explains the urgent need for standardisation in this field using a common reference preparation, e.g. the WHO International Standard (IS), in combination with common unitage reporting, e.g. international units associated with the WHO IS [21]. Finally, Cq values of our panel members are not directly comparable to those of clinical specimens in other studies: we quantified the panel members by pipetting an aliquot into the amplification reaction while viral RNA in clinical specimens is measured after its elution from swabs, with probable swab-dependant retention of viral compounds, as described in Puyskens et al [9].

We recognise as potential limitation that clinical specimens are defined according time point of symptom onset and may not necessarily reflect the same viral load pattern as in our panel. We followed routine use of the tests as far as possible, including pre-analytical steps such as antigen absorption using the test-specific swabs, and subsequent elution into the test-specific buffer. Although this procedure does not follow the IFU exactly, we estimate that it is very close to the routine steps prescribed in the IFU of each test for processing clinical specimens. The vast majority (79%) of Ag RDT included in our study showed sufficient sensitivity according to our criteria. Nevertheless, the results showed a wide range of varying sensitivity. There were few tests with high and many tests with sufficient sensitivity, but also quite a few tests (21%) that did not meet the minimum criterion. Our study shows that the majority of SARS-CoV-2 Ag RDT correctly identify high viral loads of Cq ≤ 25 (> 10^6^ virus RNA copies/mL) in samples from the respiratory tract with a sensitivity of > 75%, supporting their use in the early symptomatic phase. However, although sensitivity declined with Cq > 25, there were few SARS-CoV-2 Ag RDT (4/122; 3.3%) with highest sensitivity: 97.5% for Cq < 30 or up to 86% for the complete Cq range (Cq 17–36). 

There are scientific publications of further independent head-to-head evaluations for SARS-CoV-2 Ag RDT which, at the time of writing this manuscript, were limited to the comparison of only few tests [22-28]. Respective conclusions based on clinical specimens are widely consistent with our results, and the sensitivity ranking of different tests was often in line with our evaluation panel. For a valid comparison between different RDT, it is essential to follow the instructions for use, including the use of the swabs provided with the specific RDT, potentially impacting the release of virus compounds into the elution buffer (see also [9]). This precondition is not always fulfilled by studies comparing different RDT. 

Since most of the SARS-CoV-2 Ag RDT offered in Europe are provided without a read-out device, visual interpretation of test results is indispensable. We would like to emphasise that few discrepant tests results obtained by two experienced laboratory technicians were reported. These equivocal results were ultimately interpreted as reactive, in favour of the tests under investigation. However, visual read-out and subjective interpretation of faint test lines, potentially caused by borderline concentration of the analyte, presents a challenge for less experienced users, e.g. lay persons using Ag RDT as self-tests.

A limitation of this study is its spot check nature since it cannot address variations between different batches of the same product, or variations between different test locations (see also [9]).

## Conclusion

By using the same panel for a large number of different SARS-CoV-2 Ag RDT, we were able to evaluate the comparative performance of the different tests under the same conditions. The evaluation panel proved to be of appropriate design for sensitivity differentiation of SARS-CoV-2 Ag RDT, distinguishing better performing from less suitable tests. The continuation of the comparative evaluation is needed to cope with the rapidly growing market of SARS-CoV-2 Ag RDT. Since the panel is now close to exhaustion, we will continue the evaluation with a new set of samples with similar features, calibrated against the current panel. Although the study has not been performed with individual clinical samples, the respective limitation may be small because of the concept to use pooled specimens from clinical samples; we are confident that the results reflect well pre-analytical and analytical features of the RDT.

## Supplementary Data

950000 Supplement
