# Author's correction for Euro Surveill. 2021;26(44)

**DOI:** 10.2807/1560-7917.ES.2021.27.3.210120c

**Published:** 2022-01-20

**Authors:** Eurosurveillance editorial team

**Affiliations:** 1 European Centre for Disease Prevention and Control (ECDC), Stockholm, Sweden

In the article ‘Comparative sensitivity evaluation for 122 CE-marked rapid diagnostic tests for SARS-CoV-2 antigen, Germany, September 2020 to April 2021’ by Scheiblauer et al., published on 4 November 2021, a footnote was added in Table 2 further explaining the reason for the results (originally labelled as 0% sensitivity) obtained for tests 107 and 109 which showed false-positive background activity to all panel samples and to all negative controls, in contrast to the negative results obtained for tests 99 and 118. These changes were made on request of the authors on 22 January 2022.

