# Supplementary material for: Comparative sensitivity evaluation for 122 CE-marked rapid diagnostic tests for SARS-CoV-2 antigen, Germany, September 2020 to April 2021
Source: Euro Surveill. 2021 Nov 4;26(44):2100441. doi: 10.2807/1560-7917.ES.2021.26.44.2100441 (PMC8569926; doi:10.2807/1560-7917.ES.2021.26.44.2100441)
Supplement: Supplement [file 21-00441_NUEBLING_Supplement.pdf]

This supplementary material is hosted by Eurosurveillance as supporting information alongside the article “Comparative sensitivity evaluation for 122 CE-marked rapid diagnostic tests for SARS-CoV-2 antigen, Germany, September 2020 to April 2021”, on behalf of the authors, who remain responsible for the accuracy and appropriateness of the content. The same standards for ethics, copyright, attributions and permissions as for the article apply. Supplements are not edited by Eurosurveillance and the journal is not responsible for the maintenance of any links or email addresses provided therein.

Design and manufacture of the evaluation panel

To enable the systematic and comparable decentralized evaluation of numerous RDTs, a panel of 50 samples was compiled by pooling a total of approximately 500 upper respiratory specimens from symptomatic patients which had been collected between March and September 2020 (Panel 1V1). For pooling, dry swabs resuspended in PBS were used, with only a low number of swabs obtained in medium, resulting in a final concentration of medium in pools of ≤20% for each pool, ≤10% for 38 pools and 0% medium for 25 pools.

Subsequently, up to ten respiratory specimens obtained for routine diagnostics with different virus loads as determined by real-time PCR were pooled and diluted to a defined RNA load in PBS. Pools were frozen at -80 °C. Real-time PCR was applied to determine the RNA load per pool. *In vitro* RNA (provided by the World Health Organisation WHO) as well as the quantitative reference material provided by INSTAND were used for quantification (https://www.instand-ev.de). Finally, the panel covered a range of SARS-CoV-2 RNA from $1.1x10^9$ genomes per mL down to 420 genomes per mL. When Panel 1V1 was used up, new pools were generated by diluting the same samples as for Panel 1V1 (except for 4 pools 1–4 that had to be constituted from new clinical specimens collected between October 2020 and January 2021), resulting in comparable virus loads as determined by real-time PCR, and the panel was labelled Panel 1V2. Additionally, panel 1V1 was compared to Panel 1V2 with RDT #3 and RDT #31 and showed identical results. Panel 1V1 and Panel 1V2 have been extensively used to evaluate the sensitivity of 122 RDTs. As negative control respiratory specimens obtained by swabbing SARS-CoV-2-negative patients were pooled.

Previous studies revealed that a minimal RNA genome copy number of 10^6 genome copies per mL of specimen represents an amount of infectious virus particles which is required for

successful virus propagation in cell culture. To correlate the pools to potential infectivity by a specimen, we subjected the pools with ≥10^6 genome copies per mL, corresponding to a Cq value <25, to cell culture. Confirmation of replication-competent SARS-CoV-2 was achieved by inoculation of VeroE6 cells with the respective pools. Pools containing infectious SARS-CoV-2 were subsequently titrated on VeroE6 cells. However, even if pools containing higher amounts of SARS-CoV-2 RNA showed generally higher titres than those with lower genome numbers, we observed no significant correlation between the genome load and the titre (data not shown).

The specifications of the 50 pools are listed in the Table below; pools allowing SARS-CoV-2 propagation are marked in bold.

Table: Characteristics of the 50 pools constituting Panel 1V1 and Panel 1V2

**Panel 1V1**			**Panel 1V2**		
**Pool No.**	**Cq/5 µL of RNA**	**RNA copies subjected to test**	**Pool No.**	**Cq/5 µL of RNA**	**RNA copies subjected to test**
1	**17.55**	1.1E+07	1	**17.31**	1.31E+07
2	**20.54**	1.4E+06	2	**19.08**	3.87E+06
3	20.38	1.6E+06	3	**19.62**	2.67E+06
4	**20.98**	1.0E+06	4	**20.61**	1.35E+06
5	**20.28**	1.7E+06	5	**20.60**	1.36E+06
6	**20.20**	1.8E+06	6	21.21	8.96E+05
7	**21.71**	6.4E+05	7	22.15	4.70E+05
8	21.95	5.4E+05	8	22.32	4.18E+05

9	22.14	4.7E+05	9	23.13	2.39E+05
10	22.88	2.8E+05	10	23.21	2.27E+05
11	22.34	4.1E+05	11	23.13	2.27E+05
12	**21.82**	5.9E+05	12	22.12	4.79E+05
13	23.32	2.1E+05	13	25.29	5.42E+04
14	24.28	1.1E+05	14	24.97	6.76E+04
15	**24.14**	1.2E+05	15	24.38	1.01E+05
16	22.55	3.6E+05	16	22.88	2.84E+05
17	24.00	1.3E+05	17	24.81	7.54E+04
18	25.30	5.4E+04	18	28.33	6.71E+03
19	25.50	4.7E+04	19	25.45	2.39E+05
20	26.27	2.8E+04	20	29.46	2.27E+05
21	25.54	4.6E+04	21	25.95	2.27E+05
22	25.87	3.7E+04	22	27.42	4.79E+05
23	**24.04**	1.3E+05	23	24.45	5.42E+04
24	25.24	5.6E+04	24	25.20	6.76E+04
25	29.70	2.6E+03	25	25.07	1.01E+05
26	25.47	4.8E+04	26	26.32	2.84E+05
27	25.14	6.0E+04	27	26.12	7.54E+04
28	27.14	1.5E+04	28	27.41	6.71E+03
29	27.15	1.5E+04	29	27.34	1.33E+04
30	28.86	4.7E+03	30	27.24	1.42E+04
31	25.27	5.5E+04	31	26.24	1.42E+04
32	26.44	2.5E+04	32	26.64	2.14E+04

33	28.96	4.4E+03	33	28.92	4.47E+03
34	27.89	9.1E+03	34	27.82	9.53E+03
35	27.04	1.6E+04	35	26.66	2.12E+04
36	28.13	7.7E+03	36	27.05	1.62E+04
37	30.54	1.5E+03	37	30.13	1.95E+03
38	28.14	7.6E+03	38	29.36	3.31E+03
39	29.76	2.5E+03	39	30.12	1.96E+03
40	27.65	1.1E+04	40	28.19	7.39E+03
41	30.13	1.9E+03	41	30.14	7.39E+03
42	28.43	6.2E+03	42	29.48	3.04E+03
43	31.05	1.0E+03	43	31.61	7.04E+02
44	29.24	3.6E+03	44	29.51	2.98E+03
45	30.10	2.0E+03	45	31.19	9.40E+02
46	31.54	7.4E+02	46	31.34	8.48E+02
47	35.19	6.0E+01	47	34.55	9.34E+01
48	32.06	5.2E+02	48	31.19	9.40E+02
49	35.22	5.9E+01	49	36.04	3.35E+01
50	36.36	2.7E+01	50	35.83	3.87E+01

Quantification Cycle values (Cq values) given in bold indicate that SARS-CoV-2 could be propagated in cell culture.

Source: Puyskens et al (2021). Establishment of a specimen panel for the decentralised technical evaluation of the sensitivity of 31 rapid diagnostic tests for SARS-CoV-2 antigen, Germany, September 2020 to April 2021. Eurosurveillance

Figure 1
Comparative evaluation results of SARS-CoV-2 antigen RDT passing the sensitivity criteria

				1	2	3	4	5	6	7	8	9	10	11	12	13	14	15	16	17
			**Manufacturer**	Abbott Rapid Diagnostics Jena GmbH	ACON Biotech (Hangzhou) Co., Ltd	Aesku Diagnostics GmbH	Affimedix	Amazing Biotech (Shanghai) Co., Ltd	Ameda Labordiagnostik GmbH	AmonMed (Xiamen) Biotechnology Co.,Ltd.	Anbio (Xiamen) Biotechnology Co., Ltd	Anhui Deepblue Medical Technology Co., Ltd.	ASAN PHARM.CO.,LTD.	Atlas Link Technology Co.,Ltd.	Avalun	AXIOM Gesellschaft für Diagnostica und Biochemica mbH	Azure Biotech Inc.	Becton Dickinson	Beijing Beier Bioengineering Co., Ltd.	Beijing Hotgen Biotech Co., Ltd.
			**Test name**	Panbio™ Covid-19 Ag Rapid Test Device	Flowflex SARS-CoV-2-Antigenschnelltest (Nasopharynxtupfer)	Aesku Rapid SARS-CoV-2 Rapid Test	TestNOW® - COVID-19 Antigen	CoroVisio Covid-19 Ag Versieglungsröhrchen Teststreifen (Kolloidales Gold)	AMP Rapid Test SARS-CoV-2 Ag	COVID-19 Antigen Rapid Test Kit (Colloidal Gold)	Rapid Covid-19 Antigen Test (Colloidal Gold)	COVID-19 (SARS CoV-2) Antigen Test Kit (Colloidal Gold)	Asan Easy Test COVID-19 Ag	Nova Test SARS-CoV-2 Antigen Rapid Test Kit	Ksmart® SARS-COV2 Antigen Rapid Test	Axiom Diagnostics COVID-19 Ag Schnelltest	Dia Sure Covid-19 Antigen Rapid Test Device (Nasopharyngeal/ Oropharyngeal Swab)	BD Veritor™ System for Rapid Detection of SARS-CoV-2	Covid-19 Antigen Schnelltest	Neuartiges Coronavirus (2019-nCoV)-Antigentest (Kolloidales Gold)
**Panel 1 V1**		**Panel 1 V2**		Panel 1 V1	Panel 1 V2	Panel 1 V2	Panel 1 V1	Panel 1 V2	Panel 1 V2	Panel 1 V2	Panel 1 V2	Panel 1 V2	Panel 1 V2	Panel 1 V2	Panel 1 V2	Panel 1 V2	Panel 1 V2	Panel 1 V1	Panel 1 V1	Panel 1 V2
**Pool Nr.**	**Cq**	**Pool Nr.**	**Cq**																	
1	17,55	1	17,31	1	1	1	1	1	1	1	1	1	1	1	1	1	1	1	1	1
6	20,20	2	19,08	1	1	1	1	1	1	1	1	1	1	1	1	1	1	1	1	1
5	20,28	3	19,62	1	1	1	1	1	1	1	1	1	1	1	1	1	1	1	1	1
3	20,38	5	20,60	1	1	1	1	1	1	1	1	1	1	1	1	1	1	1	1	1
2	20,54	4	20,61	1	1	1	1	1	1	1	1	1	1	1	1	1	1	1	1	1
4	20,98	6	21,21	1	1	1	1	1	1	1	1	1	1	1	1	1	1	1	1	1
7	21,71	12	22,12	1	1	1	1	0	1	1	1	1	1	1	1	1	1	1	1	1
12	21,82	7	22,15	1	1	1	1	1	1	1	1	1	1	1	1	1	1	1	1	1
8	21,95	8	22,32	1	1	1	1	0	1	1	1	1	1	1	1	1	1	1	1	1
9	22,14	16	22,88	1	1	1	1	1	1	1	1	1	1	1	1	1	1	1	1	1
11	22,34	9	23,13	1	1	1	1	1	1	1	1	1	1	1	1	1	1	0	1	1
16	22,55	11	23,13	1	1	0	1	1	1	1	1	1	1	1	0	1	1	1	1	1
10	22,88	10	23,21	1	1	1	1	1	1	1	1	1	1	1	1	1	1	1	0	1
13	23,32	15	24,38	1	1	0	1	0	1	1	1	1	1	1	1	1	1	1	1	1
17	24,00	23	24,45	1	1	0	1	0	1	1	1	1	1	1	1	1	0	1	1	1
23	24,04	17	24,81	1	0	1	1	1	1	1	1	1	1	1	1	1	0	0	0	1
15	24,14	14	24,97	1	1	1	1	1	1	1	1	1	1	1	1	1	0	0	0	1
14	24,28	25	25,07	1	0	0	1	0	1	1	1	1	1	1	1	1	0	1	0	1
27	25,14	24	25,20	1	0	0	1	1	1	1	1	1	1	1	0	1	0	0	0	1
24	25,24	13	25,29	1	1	1	1	1	1	1	1	1	1	1	1	1	1	0	0	1
31	25,27	19	25,45	0	0	1	0	0	1	1	1	1	1	1	0	1	0	0	0	1
18	25,30	21	25,95	1	0	0	1	0	1	1	0	1	1	1	0	0	0	1	0	1
26	25,47	27	26,12	1	0	0	1	0	1	1	1	1	1	1	0	1	0	1	0	1
19	25,50	31	26,24	1	0	0	1	0	1	1	1	0	1	1	0	0	0	0	0	0
21	25,54	26	26,32	1	0	0	1	0	1	1	0	0	1	0	0	1	0	0	0	0
22	25,87	32	26,64	1	0	0	1	0	1	1	0	0	0	0	0	0	0	0	0	0
20	26,27	35	26,66	1	0	0	1	0	1	1	1	0	1	1	0	0	1	0	0	1
32	26,44	36	27,05	0	0	0	0	0	1	1	1	0	1	1	0	0	1	0	0	0
35	27,04	30	27,24	1	0	0	1	0	1	1	1	0	1	0	0	1	0	0	0	1
28	27,14	29	27,34	0	0	0	0	0	1	1	0	0	1	1	0	1	0	0	0	0
29	27,15	28	27,41	1	0	0	0	0	1	1	0	0	0	0	0	1	0	0	0	0
40	27,65	22	27,42	0	0	1	0	0	1	1	1	1	1	1	0	1	0	0	0	1
34	27,89	34	27,82	1	0	0	0	0	0	0	0	0	0	0	0	0	0	0	0	0
36	28,13	40	28,19	1	0	0	1	0	0	0	0	0	0	0	0	0	0	0	0	0
38	28,14	18	28,33	1	0	1	0	0	1	1	1	1	1	1	1	1	0	0	0	1
42	28,43	33	28,92	0	0	0	0	0	0	1	0	0	0	0	0	0	0	0	0	0
30	28,86	38	29,36	1	0	0	0	0	1	1	0	0	1	1	0	0	0	0	0	0
33	28,96	20	29,46	0	0	0	0	0	1	1	1	1	1	1	0	1	0	0	0	1
44	29,24	42	29,48	0	0	0	0	0	0	1	0	0	0	0	0	0	0	0	0	0
25	29,70	44	29,51	0	0	0	1	0	0	0	0	0	0	0	0	0	0	0	0	0
39	29,76	39	30,12	0	0	0	0	0	0	1	0	0	0	0	0	0	0	0	0	0
45	30,10	37	30,13	0	0	0	0	0	0	1	0	0	0	0	0	0	0	0	0	0
41	30,13	41	30,14	0	0	0	0	0	0	0	0	0	0	0	0	0	0	0	0	0
37	30,54	45	31,19	0	0	0	0	0	0	0	0	0	0	0	0	0	0	0	0	0
43	31,05	48	31,19	0	0	0	0	0	0	0	0	0	0	0	0	0	0	0	0	0
46	31,54	46	31,34	0	0	0	0	0	0	1	0	0	0	0	0	0	1	0	0	0
48	32,06	43	31,61	0	0	0	0	0	0	0	0	0	0	0	0	0	1	0	0	0
47	35,19	47	34,55	0	0	0	0	0	0	0	0	0	0	0	0	0	0	0	0	0
49	35,22	50	35,83	0	0	0	0	0	0	0	0	0	0	0	0	0	0	0	0	0
50	36,36	49	36,04	0	0	0	0	0	0	0	0	0	0	0	0	0	0	0	0	0

				18	19	20	21	22	23	24	25	26	27	28	29	30	31	32	33	34
			**Manufacturer**	Beijing Lepu Medical Technology Co., Ltd	Beijing Tigsun Diagnostics Co.;Ltd.	BIOMERICA Inc.	BIONOTE	BioRepair GmbH	BIOSYNEX SWISS SA	BTNX, Inc. (Biotrend Chemikalien GmbH)	Chil Tibbi Mal. San. Tic. Ltd. Şti	Core Technology Co., Ltd.	DNA Diagnostic A/S.	Edinburgh Genetics Limited	Eurobio Scientific	Fujirebio Inc. (Mast Diagnostica GmbH)	Genrui Biotech Inc.	GenSure Biotech Inc.	Getein Biotech, Inc.	Green Cross Medical Science Corp. (Weko Pharma GmbH)
			**Test name**	SARS-CoV-2 Antigen Rapid Test Kit	Tigsun COVID-19 Saliva Antigen Rapid Test	COVID-19-Antigen-Schnelltest (Nasopharyngeal-Abstrich)	NowCheck® COVID-19 Ag Test	Covid 19 Antigen Schnelltest	BIOSYNEX COVID-19 Ag BSS	Rapid Response COVID-19 Rapid Test Device	COVID-19 Antigen Schnell Test (Nasopharyngeal / Oropharyngeal Tupfer - Kassette)	Canea COVID-19 Antigen Schnelltest	Covid-19 Antigen Detection Kit	Edinburgh Genetics ActivXpress+ COVID-19 Antigen Complete Testing Kit	EBS SARS-CoV-2 Ag Rapid Test	ESPLINE® SARS-CoV-2	Genrui SARS-CoV-2 Antigen Test Kit (Colloidal Gold)	GensureTM COVID-19 Antigen Rapid Test Kit	One Step Test for SARS-CoV-2 Antigen (Colloidal Gold)	Genedia W Covid-19 Ag
**Panel 1 V1**		**Panel 1 V2**		Panel 1 V2	Panel 1 V2	Panel 1 V2	Panel 1 V1	Panel 1 V2	Panel 1 V1	Panel 1 V2	Panel 1 V2	Panel 1 V2	Panel 1 V2	Panel 1 V2	Panel 1 V2	Panel 1 V1	Panel 1 V2	Panel 1 V2	Panel 1 V2	Panel 1 V1
**Pool Nr.**	**Cq**	**Pool Nr.**	**Cq**																	
1	17,55	1	17,31	1	1	1	1	1	1	1	1	1	1	1	1	1	1	1	1	1
6	20,20	2	19,08	1	1	1	1	1	1	1	1	1	1	1	1	1	1	1	1	1
5	20,28	3	19,62	1	1	1	1	1	1	1	1	1	1	1	1	1	1	1	1	1
3	20,38	5	20,60	1	1	1	1	1	1	1	1	1	1	1	1	1	1	1	1	1
2	20,54	4	20,61	1	1	1	1	1	1	1	1	1	1	1	1	1	1	1	1	1
4	20,98	6	21,21	1	1	1	1	1	1	1	1	1	1	1	1	1	1	1	1	1
7	21,71	12	22,12	1	1	1	1	1	1	1	1	1	1	1	1	1	1	1	1	1
12	21,82	7	22,15	1	1	1	1	1	1	1	1	1	1	1	1	1	1	1	1	1
8	21,95	8	22,32	1	1	1	1	1	1	1	1	1	1	1	1	1	1	1	1	1
9	22,14	16	22,88	1	1	1	1	1	1	1	1	1	1	1	1	1	1	1	1	1
11	22,34	9	23,13	1	1	1	1	1	1	1	1	1	1	1	1	1	1	1	1	1
16	22,55	11	23,13	1	1	1	1	1	1	1	1	1	1	1	1	1	1	1	1	1
10	22,88	10	23,21	1	1	1	1	1	1	1	1	1	1	1	1	1	1	1	1	1
13	23,32	15	24,38	1	1	1	1	1	1	0	1	0	1	1	1	1	1	1	1	1
17	24,00	23	24,45	1	1	1	1	1	1	1	1	0	1	1	0	1	0	0	1	1
23	24,04	17	24,81	1	1	1	1	1	1	1	1	1	1	1	1	1	1	1	1	0
15	24,14	14	24,97	1	1	1	1	1	1	1	1	1	1	1	1	1	1	1	1	0
14	24,28	25	25,07	1	1	1	1	1	1	0	1	1	1	1	1	1	1	1	1	0
27	25,14	24	25,20	0	1	1	1	1	1	0	1	0	1	1	0	1	1	0	1	0
24	25,24	13	25,29	1	1	1	1	1	1	1	1	1	1	1	1	0	1	1	1	0
31	25,27	19	25,45	1	1	1	1	1	1	0	1	1	1	1	1	0	1	0	1	0
18	25,30	21	25,95	0	1	0	1	1	1	0	1	0	1	0	0	1	1	0	1	1
26	25,47	27	26,12	0	1	0	1	1	1	0	1	1	1	1	1	1	1	0	1	1
19	25,50	31	26,24	0	1	0	1	1	1	0	0	0	0	0	0	1	0	0	1	0
21	25,54	26	26,32	0	1	1	1	1	1	0	1	0	0	0	1	0	0	0	1	0
22	25,87	32	26,64	0	1	0	1	1	1	0	1	0	0	0	1	0	1	0	1	0
20	26,27	35	26,66	0	1	0	1	1	1	0	0	0	0	0	0	0	1	0	1	0
32	26,44	36	27,05	0	1	0	0	1	1	1	1	0	0	0	0	0	0	0	1	0
35	27,04	30	27,24	0	1	0	1	1	1	0	1	0	0	0	0	1	0	0	1	0
28	27,14	29	27,34	1	1	0	0	1	0	0	1	0	0	0	0	0	1	0	1	0
29	27,15	28	27,41	0	1	0	1	1	1	1	0	0	0	0	0	0	0	0	1	0
40	27,65	22	27,42	1	1	0	0	1	0	0	1	1	1	1	0	0	1	0	1	0
34	27,89	34	27,82	0	1	0	1	0	1	0	0	0	0	0	0	0	1	0	1	0
36	28,13	40	28,19	0	0	0	1	0	1	0	0	0	0	0	0	0	N/A	0	0	0
38	28,14	18	28,33	1	1	1	1	1	1	0	1	1	1	1	1	0	1	1	1	0
42	28,43	33	28,92	0	1	0	0	0	0	0	0	0	0	0	0	0	N/A	0	0	0
30	28,86	38	29,36	0	1	0	1	1	1	0	0	0	0	0	0	0	0	0	1	0
33	28,96	20	29,46	0	1	1	0	1	0	0	1	0	1	1	1	0	1	0	1	0
44	29,24	42	29,48	0	0	0	0	0	0	0	0	0	0	0	0	0	0	0	0	0
25	29,70	44	29,51	0	0	0	0	0	1	0	0	0	0	0	0	0	0	0	0	0
39	29,76	39	30,12	0	0	0	0	0	1	0	0	0	0	0	0	0	0	0	0	0
45	30,10	37	30,13	0	1	0	0	0	0	0	0	0	0	0	0	0	0	0	0	0
41	30,13	41	30,14	0	0	0	0	0	1	0	0	0	0	0	0	0	0	0	0	0
37	30,54	45	31,19	0	0	0	0	0	0	0	0	0	0	0	0	0	0	0	0	0
43	31,05	48	31,19	0	1	0	0	0	0	0	0	0	0	0	0	0	0	0	0	0
46	31,54	46	31,34	0	1	0	0	0	0	1	0	0	0	0	0	0	0	0	0	0
48	32,06	43	31,61	invalid	0	0	0	0	0	0	0	0	0	0	0	0	0	0	0	0
47	35,19	47	34,55	0	0	0	0	0	0	0	0	0	0	0	0	0	0	0	0	0
49	35,22	50	35,83	0	0	0	0	0	0	0	0	0	0	0	0	0	0	0	0	0
50	36,36	49	36,04	0	0	0	0	0	0	0	0	0	0	0	0	0	0	0	0	0

				35	36	37	38	39	40	41	42	43	44	45	46	47	48	49	50	51
			**Manufacturer**	Guangdong Hecin Scientific,Inc.	Guangdong Wesail Biotech Co., Ltd.	Guangzhou Wondfo Biotech Co. Ltd	Hangzhou Clongene Biotech Co., Ltd.	Hangzhou Immuno Biotech Co.,Ltd.	Hangzhou Laihe Biotech Co., Ltd. (Lissner Qi GmbH)	Hangzhou Lysun Biotechnology Co., Ltd.	Hangzhou Testsea Biotechnology Co., Ltd	Humasis Co., Ltd.	IVC Pragen Healthcare	Jiangsu Diagnostics Biotechnology Co., Ltd	Jiangsu Medomics Medical Technology Co., Ltd	Joinstar Biomedical Technology Co., Ltd (CIV care impuls Vertrieb)	Labnovation Technologies, Inc.	Lumigenex (Suzhou) Co., Ltd.	LumiQuick Diagnostics Inc.	LumiraDX
			**Test name**	2019-nCoV Antigen Test Kit (colloidal gold method)	COVID-19 Ag Test Kit	Wondfo SARS-CoV-2 Antigen Test (Lateral Flow Method)	Clungene COVID-19 Antigen Rapid Test	IMMUNOBIO SARS-CoV-2 Antigen-Schnelltest (COVID-19 Ag)	Lyher Novel Coronavirus (COVID-19) Antigen Test Kit (Colloidal Gold)	Lysun COVID-19 Antigen Rapid Test Device (Colloidal Gold)	Testsealabs® Rapid Test Kit COVID-19 Antigen Test Cassette	COVID-19 Ag Test	GenBody COVID-19 Ag	COVID-19 Antigen Rapid Test Cassette (Colloidal Gold)	SARS-CoV-2-Antigen-Testkit (LFIA)	COVID-19 Antigen Schnelltest (Colloidal Gold)	Labnovation SARS-CoV-2 Antigen Rapid Test Kit (Immunochromatography)	PocRoc SARS-CoV-2, Antigen Schnelltest Set (Kolloidales Gold)	QuickProfile Covid-19 Antigen Test Card	LumiraDx SARS-CoV-2 Ag Test
**Panel 1 V1**		**Panel 1 V2**		Panel 1 V2	Panel 1 V1	Panel 1 V2	Panel 1 V1	Panel 1 V2	Panel 1 V1	Panel 1 V2	Panel 1 V2	Panel 1 V2	Panel 1 V1	Panel 1 V2	Panel 1 V2	Panel 1 V1	Panel 1 V2	Panel 1 V2	Panel 1 V2	Panel 1 V1
**Pool Nr.**	**Cq**	**Pool Nr.**	**Cq**																	
1	17,55	1	17,31	1	1	1	1	1	1	1	1	1	1	1	1	1	1	1	1	1
6	20,20	2	19,08	1	1	1	1	1	1	1	1	1	1	1	1	1	1	1	1	1
5	20,28	3	19,62	1	1	1	1	1	1	1	1	1	1	1	1	1	1	1	1	1
3	20,38	5	20,60	1	1	1	1	1	1	1	1	1	1	1	1	1	1	1	1	1
2	20,54	4	20,61	1	1	1	1	1	1	1	1	1	1	1	1	1	1	1	1	1
4	20,98	6	21,21	1	1	1	1	1	1	1	1	1	1	1	1	1	1	1	1	1
7	21,71	12	22,12	1	1	1	1	1	1	1	1	1	1	1	1	1	1	1	1	1
12	21,82	7	22,15	1	1	1	1	1	1	1	1	1	1	1	1	1	1	1	1	1
8	21,95	8	22,32	1	1	1	1	1	1	1	1	1	1	1	1	1	1	1	1	1
9	22,14	16	22,88	1	1	1	1	1	1	1	1	1	1	1	1	1	1	1	1	1
11	22,34	9	23,13	1	1	1	1	1	1	1	1	1	1	1	1	1	1	1	1	1
16	22,55	11	23,13	0	1	1	1	0	1	1	1	0	1	1	1	1	1	1	1	1
10	22,88	10	23,21	1	1	1	1	1	1	1	1	1	1	1	1	1	1	1	1	1
13	23,32	15	24,38	0	1	1	1	1	1	1	1	1	1	1	1	1	1	1	1	1
17	24,00	23	24,45	0	1	0	1	0	1	1	1	0	1	1	0	1	0	1	1	1
23	24,04	17	24,81	1	1	1	0	1	0	1	1	1	0	1	1	1	1	1	1	1
15	24,14	14	24,97	1	1	0	1	1	1	1	1	1	1	1	1	1	1	1	1	1
14	24,28	25	25,07	0	1	0	1	0	1	1	1	1	1	1	0	1	0	1	1	1
27	25,14	24	25,20	0	1	0	1	0	0	1	1	0	1	1	0	1	0	1	1	1
24	25,24	13	25,29	1	1	0	0	1	0	1	1	1	0	1	1	1	1	1	1	0
31	25,27	19	25,45	0	0	0	0	1	0	1	1	0	0	1	1	1	1	1	1	0
18	25,30	21	25,95	0	1	0	1	0	1	1	0	1	1	1	0	1	0	0	1	1
26	25,47	27	26,12	0	1	0	1	0	1	1	1	1	1	1	0	1	0	1	1	1
19	25,50	31	26,24	0	1	0	1	0	0	1	0	0	1	1	0	1	0	1	1	1
21	25,54	26	26,32	0	1	0	0	0	0	1	0	0	0	1	0	1	0	1	1	0
22	25,87	32	26,64	0	1	0	1	0	1	1	0	0	1	1	0	1	0	1	1	1
20	26,27	35	26,66	0	1	0	1	0	0	1	1	0	0	1	0	1	0	1	1	1
32	26,44	36	27,05	0	0	0	0	0	0	1	0	0	0	1	0	1	0	1	1	1
35	27,04	30	27,24	0	1	0	0	0	0	1	1	0	1	1	0	1	0	0	1	1
28	27,14	29	27,34	0	0	0	0	0	0	1	1	0	0	1	0	0	0	1	1	0
29	27,15	28	27,41	0	1	0	1	0	0	1	0	0	0	1	0	0	0	0	1	1
40	27,65	22	27,42	0	0	0	0	0	0	1	1	0	0	1	1	0	1	1	1	0
34	27,89	34	27,82	0	0	0	0	0	0	0	0	0	0	0	0	0	0	0	1	0
36	28,13	40	28,19	0	1	0	0	0	0	0	0	0	0	0	0	1	N/A	0	0	1
38	28,14	18	28,33	1	0	0	0	1	0	1	1	1	0	1	1	0	1	1	1	0
42	28,43	33	28,92	0	0	0	0	0	0	0	0	0	0	0	0	0	N/A	0	0	0
30	28,86	38	29,36	0	1	0	0	0	0	1	0	0	0	1	0	1	0	1	1	1
33	28,96	20	29,46	1	0	0	0	0	0	1	1	0	0	1	1	0	0	1	1	0
44	29,24	42	29,48	0	0	0	0	0	0	0	0	0	0	0	0	0	0	0	1	0
25	29,70	44	29,51	0	0	0	1	0	0	0	0	0	0	0	0	1	0	0	1	1
39	29,76	39	30,12	0	0	0	0	0	0	0	0	0	0	0	0	0	0	0	1	0
45	30,10	37	30,13	0	0	0	0	0	0	0	0	0	0	0	0	0	0	0	0	0
41	30,13	41	30,14	0	0	0	0	0	0	0	0	0	0	0	0	0	0	0	0	0
37	30,54	45	31,19	0	0	0	0	0	0	0	0	0	0	0	0	0	0	0	0	0
43	31,05	48	31,19	0	0	0	0	0	0	0	0	0	0	0	0	0	0	0	0	0
46	31,54	46	31,34	0	0	0	0	0	0	0	0	0	0	0	0	0	0	0	0	0
48	32,06	43	31,61	0	0	0	0	0	0	0	0	0	0	0	0	0	0	0	1	0
47	35,19	47	34,55	0	1	0	0	0	0	0	0	0	0	0	0	0	0	0	0	0
49	35,22	50	35,83	0	0	0	0	0	0	0	0	0	0	0	0	0	0	0	0	0
50	36,36	49	36,04	0	0	0	0	0	0	0	0	0	0	0	0	0	0	0	0	0

				52	53	54	55	56	57	58	59	60	61	62	63	64	65	66	67	68
			**Manufacturer**	MEDsan GmbH	Merlin Biomedical (Xiamen) Co., Ltd.	Mölab GmbH	MP Biomedicals Germany GmbH	nal von minden GmbH	NanoEntek Inc.	Nanjing Norman Biological Technology Co.,Ltd	Nantong Diagnos Biotechnology Co., Ltd.	New Gene (Hangzhou) Bioengineering Co., Ltd.	Novatech Tibbi Cihaz Ürünleri San. Ve Tic. A.S.	Oncosem Onkolojik Sistemler San. Ve Tic. A.S.	PCL, Inc.	PerGrande BioTech Development Co., Ltd.	Precision Biosensor Inc. (Axon Lab AG)	ProGnosis Biotech	Quidel Corporation	Qingdao Hightop Biotech Co., Ltd.
			**Test name**	MEDsan® SARS-CoV-2 Antigen Rapid Test	SARS-CoV-2 Antigen Rapid Test Cassette	mö-screen Corona Antigen Test	Rapid SARS-CoV-2 Antigen Test Card	NADAL® COVID-19 Ag Schnelltest	Frend™ COVID-19 Ag	Novel Coronavirus (2019-nCOV) Antigen Testing Kit (Colloidal Gold)	COVID-19 Antigen Saliva Test Kit (Colloidal Gold)	Covid-19-Antigen-Testkit	novacheck®-Ag SARS-CoV-2 Covid-19 Antigen Rapid Test	CAT Antigen Covid Rapid Test	PCL COVID19 Ag Gold Saliva	SARS-CoV-2 Antigen Detection Kit (Colloidal Gold Immuno-chromatographic Assay)	Exdia COVID-19-Ag-Test	Rapid Test Ag 2019-nCoV	Sofia SARS Antigen FIA	Hightop SARS-CoV-2 (Covid-19) Antigen Rapid Test
**Panel 1 V1**		**Panel 1 V2**		Panel 1 V1	Panel 1 V2	Panel 1 V1	Panel 1 V2	Panel 1 V1	Panel 1 V2	Panel 1 V2	Panel 1 V2	Panel 1 V2	Panel 1 V2	Panel 1 V2	Panel 1 V2	Panel 1 V2	Panel 1 V1	Panel 1 V2	Panel 1 V1	Panel 1 V2
**Pool Nr.**	**Cq**	**Pool Nr.**	**Cq**																	
1	17,55	1	17,31	1	1	1	1	1	1	1	1	1	1	1	1	1	1	1	1	1
6	20,20	2	19,08	1	1	1	1	1	1	1	1	1	1	1	1	1	1	1	1	1
5	20,28	3	19,62	1	1	1	1	1	1	1	1	1	1	1	1	1	1	1	1	1
3	20,38	5	20,60	1	1	1	1	1	1	1	1	1	1	1	1	1	1	1	1	1
2	20,54	4	20,61	1	1	1	1	1	1	1	1	1	1	1	1	1	1	1	1	1
4	20,98	6	21,21	1	1	1	1	1	1	1	1	1	1	1	1	1	1	1	1	1
7	21,71	12	22,12	1	1	1	1	1	1	1	1	1	1	1	1	1	1	1	1	1
12	21,82	7	22,15	1	1	1	1	1	1	1	1	1	1	1	1	1	1	1	1	1
8	21,95	8	22,32	1	1	1	1	1	1	1	1	1	1	1	1	1	1	1	1	1
9	22,14	16	22,88	1	1	1	1	1	1	1	1	1	1	1	1	1	1	0	1	1
11	22,34	9	23,13	1	1	1	1	1	1	1	1	1	1	1	1	1	1	1	1	1
16	22,55	11	23,13	1	1	1	1	1	1	1	1	1	1	1	1	1	1	1	1	1
10	22,88	10	23,21	1	1	1	1	1	1	1	1	1	1	1	1	1	1	1	1	1
13	23,32	15	24,38	1	1	1	1	0	0	1	1	1	1	0	1	1	1	1	1	1
17	24,00	23	24,45	1	1	1	1	1	0	0	1	1	0	1	1	1	1	1	1	1
23	24,04	17	24,81	1	1	1	1	0	1	1	1	1	1	1	1	1	1	1	0	1
15	24,14	14	24,97	1	1	1	1	1	1	1	1	1	1	1	1	1	1	1	0	1
14	24,28	25	25,07	1	1	1	1	0	0	1	1	1	1	1	1	0	1	1	1	1
27	25,14	24	25,20	1	1	1	1	0	0	0	1	1	0	1	1	0	1	1	0	1
24	25,24	13	25,29	0	1	1	1	0	1	1	1	1	1	1	1	0	1	1	0	1
31	25,27	19	25,45	0	1	0	1	0	0	1	1	1	0	1	1	0	1	1	0	1
18	25,30	21	25,95	1	1	1	1	0	0	0	1	1	0	0	1	0	1	1	1	0
26	25,47	27	26,12	1	1	1	1	1	0	1	1	1	0	1	1	0	1	0	1	1
19	25,50	31	26,24	1	1	1	0	0	0	0	0	1	0	0	0	0	1	1	0	0
21	25,54	26	26,32	1	1	1	1	1	0	0	1	1	0	0	1	1	0	1	0	0
22	25,87	32	26,64	1	1	1	0	1	0	0	0	1	0	0	0	0	1	1	0	0
20	26,27	35	26,66	1	0	1	0	0	0	0	1	1	0	0	0	0	1	0	0	1
32	26,44	36	27,05	1	0	0	1	0	0	0	0	1	0	0	0	1	0	1	0	0
35	27,04	30	27,24	1	1	1	0	0	0	0	1	1	1	0	1	0	1	1	0	0
28	27,14	29	27,34	0	0	0	0	0	0	0	0	1	0	0	1	0	0	0	0	1
29	27,15	28	27,41	0	1	1	0	0	0	0	0	1	0	0	0	1	1	1	0	0
40	27,65	22	27,42	0	1	0	1	0	0	1	1	1	1	0	1	0	0	1	0	1
34	27,89	34	27,82	0	1	0	0	0	0	0	0	1	0	0	0	0	0	0	0	0
36	28,13	40	28,19	0	0	0	0	0	0	0	0	0	0	0	0	0	1	0	0	0
38	28,14	18	28,33	1	1	0	1	0	1	1	1	1	1	1	1	0	1	1	0	1
42	28,43	33	28,92	1	0	0	0	0	0	0	0	0	0	0	0	1	0	1	0	0
30	28,86	38	29,36	0	0	0	0	0	0	0	1	1	0	0	0	0	1	0	0	0
33	28,96	20	29,46	0	1	0	0	0	0	0	1	1	0	0	1	0	0	1	0	1
44	29,24	42	29,48	0	0	0	0	0	0	0	0	1	0	0	0	0	0	0	0	0
25	29,70	44	29,51	0	0	1	0	0	0	0	0	0	0	0	0	0	1	0	0	0
39	29,76	39	30,12	0	0	0	0	0	0	0	0	0	0	0	0	0	0	0	0	0
45	30,10	37	30,13	0	0	0	0	0	0	0	0	0	0	0	0	0	0	1	0	0
41	30,13	41	30,14	0	0	0	0	0	0	0	0	1	0	0	0	0	0	0	0	0
37	30,54	45	31,19	0	0	0	0	0	0	0	0	0	0	0	0	0	0	0	0	0
43	31,05	48	31,19	0	0	0	0	0	0	0	0	0	0	0	0	0	0	0	0	0
46	31,54	46	31,34	0	0	0	0	0	0	0	0	0	0	0	0	0	0	0	0	0
48	32,06	43	31,61	0	0	0	0	0	0	0	0	1	0	0	0	0	0	0	0	0
47	35,19	47	34,55	0	0	0	0	0	0	0	0	0	0	0	0	0	0	0	0	0
49	35,22	50	35,83	0	0	0	0	0	0	0	0	0	0	0	0	0	0	0	0	0
50	36,36	49	36,04	0	0	0	0	0	0	0	0	0	0	0	0	0	0	0	0	0

				69	70	71	72	73	74	75	76	77	78	79	80	81	82	83	84	85
			**Manufacturer**	R-Biopharm AG	Safecare Biotech Hangzhou Co., Ltd.	Salofa OY	ScheBo Biotech AG	SD BIOSENSOR (Roche Diagnostics GmbH)	SD BIOSENSOR	SD BIOSENSOR	SGA Mühendislik DAN. EG. Icve DIS.Ltd.STI	Shenzhen Lvshiyuan Biotechnology Co., Ltd.	Shenzhen Microprofit Biotech Co., Ltd.	Shenzhen Watmind Medical Co.,Ltd.	Shenzhen Watmind Medical Co.,Ltd.	Shenzhen Zhenrui Biotech co.Ltd.	Siemens Healthineers	Sugentech, Inc.	Toda Pharma	Triplex International Biosciences (China) Co., Ltd.
			**Test name**	RIDA®QUICK SARS-CoV-2 Antigen	Safecare COVID-19 Ag Rapid Test Kit (Swab)	salocor SARS-CoV-2 Antigen Rapid Test Cassette (Nasopharyngeal swab)	ScheBo SARS-CoV-2 Quick Antigen	SARS-CoV-2 Rapid Antigen Test	STANDARD™ F COVID-19 Ag FIA	STANDARD™ Q COVID-19 Ag Test	V-Chek SARS-CoV-2 Rapid Ag Test Kit (Collaidal Gold)	Green Spring SARS-CoV-2 Antigen Rapid Test Kit (Colloidal Gold)	fluorecare COVID-19 SARS-CoV-2 Spike Protein Test Kit (Colloidal Gold Chromatographic Immunoassay)	SARS-CoV-2 Ag Diagnostic Test Kit (Colloidal Gold)	SARS-CoV-2 Ag Diagnostic Test Kit (Immuno-fluorescence)	Zhenrui COVID-19 (SARS-COV-2) Antigen Test Kits	CLINITEST® Rapid COVID-19 Antigen Test	SGTi-flex COVID-19 Ag	Toda Coronadiag Ag	SARS-CoV-2 Antigen Rapid Test Kit
**Panel 1 V1**		**Panel 1 V2**		Panel 1 V1	Panel 1 V2	Panel 1 V2	Panel 1 V2	Panel 1 V1	Panel 1 V1	Panel 1 V1	Panel 1 V2	Panel 1 V2	Panel 1 V2	Panel 1 V2	Panel 1 V2	Panel 1 V2	Panel 1 V1	Panel 1 V2	Panel 1 V2	Panel 1 V2
**Pool Nr.**	**Cq**	**Pool Nr.**	**Cq**																	
1	17,55	1	17,31	1	1	1	1	1	1	1	1	1	1	1	1	1	1	1	1	1
6	20,20	2	19,08	1	1	1	1	1	1	1	1	1	1	1	1	1	1	1	1	1
5	20,28	3	19,62	1	1	1	1	1	1	1	1	1	1	1	1	1	1	1	1	1
3	20,38	5	20,60	1	1	1	1	1	1	1	1	1	1	1	1	1	1	1	1	1
2	20,54	4	20,61	1	1	1	1	1	1	1	1	1	1	1	1	1	1	1	1	1
4	20,98	6	21,21	1	1	1	1	1	1	1	1	1	1	1	1	1	1	1	1	1
7	21,71	12	22,12	1	1	1	1	1	1	1	1	1	1	1	1	1	1	1	1	1
12	21,82	7	22,15	1	1	1	1	1	1	1	1	1	1	1	1	1	1	1	1	1
8	21,95	8	22,32	1	1	1	1	1	1	1	1	1	1	1	1	1	1	1	1	1
9	22,14	16	22,88	1	1	1	1	1	1	1	1	1	1	1	1	1	1	1	1	1
11	22,34	9	23,13	1	1	1	1	1	1	1	1	1	1	1	1	1	1	1	1	1
16	22,55	11	23,13	1	1	0	1	1	1	1	1	1	1	1	1	1	1	1	1	1
10	22,88	10	23,21	1	1	1	1	1	1	1	1	1	1	1	1	1	1	1	1	1
13	23,32	15	24,38	1	1	0	1	1	1	1	1	1	1	1	1	0	1	1	1	1
17	24,00	23	24,45	1	1	0	1	1	1	1	0	1	1	1	1	0	1	1	1	1
23	24,04	17	24,81	1	1	1	1	0	1	0	1	1	1	1	1	1	1	1	1	1
15	24,14	14	24,97	1	1	1	1	0	1	0	1	1	1	1	1	0	1	1	1	1
14	24,28	25	25,07	1	1	1	1	1	1	1	1	1	1	1	1	1	1	1	1	1
27	25,14	24	25,20	0	1	0	1	1	1	1	0	1	1	1	1	0	1	1	1	1
24	25,24	13	25,29	1	1	1	1	0	1	0	1	1	1	1	1	1	1	1	1	1
31	25,27	19	25,45	0	1	0	1	0	0	0	1	1	1	1	1	0	1	1	1	1
18	25,30	21	25,95	1	1	0	1	1	1	1	N/A	1	1	1	1	0	1	0	1	1
26	25,47	27	26,12	1	1	0	1	1	1	1	0	1	1	1	1	0	1	1	1	1
19	25,50	31	26,24	0	0	0	1	1	1	1	0	1	0	1	0	0	1	1	1	1
21	25,54	26	26,32	1	1	0	1	0	1	0	0	1	0	1	1	0	1	1	1	1
22	25,87	32	26,64	0	0	0	1	1	1	1	0	1	0	1	0	0	1	0	1	1
20	26,27	35	26,66	0	1	0	1	0	1	0	0	1	0	1	1	0	1	1	1	1
32	26,44	36	27,05	0	1	0	1	0	0	0	0	1	1	1	1	0	1	1	1	1
35	27,04	30	27,24	0	1	0	1	1	1	1	0	1	0	1	1	0	1	1	1	1
28	27,14	29	27,34	0	1	0	1	0	1	0	0	1	0	1	0	0	1	1	1	1
29	27,15	28	27,41	0	0	0	1	0	1	0	0	1	0	1	0	0	1	1	1	1
40	27,65	22	27,42	0	1	0	1	0	0	0	1	1	1	1	1	0	0	1	1	1
34	27,89	34	27,82	0	0	0	1	0	1	0	0	1	1	1	0	0	1	1	1	1
36	28,13	40	28,19	0	0	N/A	1	1	1	1	N/A	1	0	1	0	0	1	0	1	0
38	28,14	18	28,33	0	1	1	1	0	1	0	1	1	1	1	1	1	1	1	1	1
42	28,43	33	28,92	0	0	0	0	0	0	0	N/A	1	0	1	0	0	1	0	1	0
30	28,86	38	29,36	0	1	0	1	0	0	0	0	1	0	1	1	0	1	1	1	1
33	28,96	20	29,46	0	0	0	1	0	0	0	1	1	1	1	1	0	1	1	1	1
44	29,24	42	29,48	0	0	0	1	0	0	0	0	1	0	1	0	0	0	0	1	1
25	29,70	44	29,51	0	0	0	0	0	1	0	0	0	0	0	0	0	1	0	0	0
39	29,76	39	30,12	0	0	0	0	0	0	0	0	1	0	0	0	0	0	0	0	0
45	30,10	37	30,13	0	0	0	0	0	0	0	0	1	1	1	0	0	0	0	1	0
41	30,13	41	30,14	0	0	0	0	0	0	0	0	0	0	1	0	0	0	0	0	1
37	30,54	45	31,19	0	0	0	1	0	0	0	N/A	0	0	0	0	0	0	0	1	0
43	31,05	48	31,19	0	0	0	0	0	0	0	0	0	0	0	0	0	0	0	0	0
46	31,54	46	31,34	0	0	0	0	0	0	0	0	1	0	0	0	0	0	0	1	0
48	32,06	43	31,61	0	0	0	0	0	0	0	0	0	0	0	0	0	0	0	0	1
47	35,19	47	34,55	0	0	0	0	0	0	0	0	1	0	0	0	0	0	0	1	0
49	35,22	50	35,83	0	0	0	0	0	0	0	0	0	0	0	0	0	0	0	0	0
50	36,36	49	36,04	0	0	0	0	0	0	0	0	0	0	0	0	0	0	0	0	0

				86	87	88	89	90	91	92	93	94	95	96
			**Manufacturer**	ulti med Products (Deutschland) GmbH	Vitrosens Biyoteknoloji Ltd. Sti	Wantai (Beijing Wantai Biological Pharmacy Enterprise Co., Ltd.)	Wuhan EasyDiagnosis Biomedicine Co., Ltd	Wuhan Life Origin Biotech Joint Stock Co., Ltd.	Wuhan UNscience Biotechnology Co., Ltd.	Xiamen Boson Biotech Co., Ltd.	Xiamen WIZ Biotech Co., Ltd.	Zet Medikal Tekstil Dis Ticaret Ltd. STI.	Zhejiang Anji Saianfu Biotech Co.,Ltd.	Zhejiang Orient Gene Biotech Co.,Ltd
			**Test name**	COVID-19 Antigen Speicheltest (Immunochromatographie)	RapidFor SARS-CoV-2 Rapid Antigen Test Colloidal Gold	SARS-CoV-2 Ag Rapid Test (FIA)	COVID-19 (SARS-CoV-2) Antigen Test Kit	SARS-CoV-2 Antigen Assay Kit (Immunochromatography)	SARS-CoV-2 Antigen Rapid Test Kit	SARS-CoV-2 Antigen Schnelltest	Wizbiotech SARS-CoV-2 Antigen Rapid Test	softec SARS COV-2 (Covid-19) Antigen Test Kit	reOpenTest COVID-19 Antigen Rapid Test (Colloidal Gold)	Coronavirus Ag Rapid Test Cassette (Swab)
**Panel 1 V1**		**Panel 1 V2**		Panel 1 V2	Panel 1 V2	Panel 1 V1	Panel 1 V2	Panel 1 V2	Panel 1 V2	Panel 1 V2	Panel 1 V2	Panel 1 V2	Panel 1 V2	Panel 1 V1
**Pool Nr.**	**Cq**	**Pool Nr.**	**Cq**											
1	17,55	1	17,31	1	1	1	1	1	1	1	1	1	1	1
6	20,20	2	19,08	1	1	1	1	1	1	1	1	1	1	1
5	20,28	3	19,62	1	1	1	1	1	1	1	1	1	1	1
3	20,38	5	20,60	1	1	1	1	1	1	1	1	1	1	1
2	20,54	4	20,61	1	1	1	1	1	1	1	1	0	1	1
4	20,98	6	21,21	1	1	1	1	1	1	1	1	1	1	1
7	21,71	12	22,12	1	1	1	1	1	1	1	1	1	1	1
12	21,82	7	22,15	1	1	1	1	1	1	1	1	1	1	1
8	21,95	8	22,32	1	1	1	1	1	1	1	1	1	1	1
9	22,14	16	22,88	1	1	1	1	1	1	1	1	1	1	1
11	22,34	9	23,13	1	1	1	1	1	1	1	1	1	1	1
16	22,55	11	23,13	1	1	1	1	1	1	1	1	1	1	1
10	22,88	10	23,21	1	1	1	1	1	1	1	1	0	1	1
13	23,32	15	24,38	1	1	1	1	1	0	1	0	1	1	1
17	24,00	23	24,45	1	1	1	1	1	0	1	0	N/A	0	1
23	24,04	17	24,81	1	1	1	1	1	1	1	1	1	1	1
15	24,14	14	24,97	1	1	1	1	1	1	1	1	1	1	1
14	24,28	25	25,07	1	1	1	1	1	1	1	0	1	1	1
27	25,14	24	25,20	1	0	1	1	1	0	1	0	1	0	1
24	25,24	13	25,29	1	1	1	1	1	1	1	1	1	1	1
31	25,27	19	25,45	1	1	1	1	1	1	1	1	0	1	1
18	25,30	21	25,95	1	0	1	1	1	0	1	0	N/A	0	1
26	25,47	27	26,12	1	1	1	1	1	0	1	0	0	1	1
19	25,50	31	26,24	1	0	1	1	1	0	0	0	invalid	0	1
21	25,54	26	26,32	1	0	1	1	0	0	1	0	1	0	1
22	25,87	32	26,64	1	0	1	1	1	0	0	0	0	0	1
20	26,27	35	26,66	1	0	1	1	1	0	0	0	0	1	1
32	26,44	36	27,05	1	0	1	1	0	0	1	0	0	0	1
35	27,04	30	27,24	1	0	1	1	1	0	0	0	0	0	1
28	27,14	29	27,34	1	0	1	1	0	0	0	0	0	0	1
29	27,15	28	27,41	1	0	1	0	0	0	0	0	0	0	1
40	27,65	22	27,42	1	1	0	1	1	0	1	0	1	1	0
34	27,89	34	27,82	1	0	1	0	0	0	0	0	0	0	1
36	28,13	40	28,19	1	0	1	0	0	0	0	0	N/A	0	1
38	28,14	18	28,33	1	1	1	1	1	1	1	1	N/A	1	1
42	28,43	33	28,92	1	0	0	0	0	0	0	0	N/A	0	1
30	28,86	38	29,36	1	0	1	1	0	0	0	0	0	0	1
33	28,96	20	29,46	1	1	0	1	1	0	0	0	0	0	1
44	29,24	42	29,48	1	0	0	0	0	0	0	0	0	0	0
25	29,70	44	29,51	0	0	1	0	0	0	0	0	0	0	1
39	29,76	39	30,12	0	0	0	0	0	0	0	0	invalid	0	0
45	30,10	37	30,13	0	0	0	0	0	0	0	0	0	0	0
41	30,13	41	30,14	1	0	0	0	0	0	0	0	0	0	0
37	30,54	45	31,19	0	0	0	0	0	0	0	0	N/A	0	0
43	31,05	48	31,19	0	0	0	0	0	0	0	0	invalid	0	0
46	31,54	46	31,34	1	0	0	0	0	0	0	0	invalid	0	0
48	32,06	43	31,61	0	0	0	0	0	0	0	0	0	0	0
47	35,19	47	34,55	0	0	0	0	0	0	0	0	0	0	0
49	35,22	50	35,83	0	0	0	0	0	0	0	0	1	0	0
50	36,36	49	36,04	0	0	0	0	0	0	0	0	invalid	0	0

Figure 2
Comparative evaluation results of SARS-CoV-2 antigen RDT missing the sensitivity criteria

				97	98	99	100	101	102	103	104	105	106	107	108	109	110	111
			**Manufacturer**	Acro Biotech Inc	Aikang Diagnostics Co., Ltd.	Beijing Savant Biotechnology Co., Ltd	CertestT Biotec S. L.	Coris Bioconcept	Hangzhou AllTest Biotech Co. Ltd.	Hangzhou Biotest Biotech Co., Ltd.	Hangzhou Genesis Biocontrol Co., Ltd	Hangzhou Realy Tech Co., Ltd.	Inzek International Trading	Joinstar Biomedical Technology Co., Ltd	Joysbio (Tianjin) Biotechnology Co., Ltd.	Lionex GmbH	Medicon Co., Ltd.	Mexacare GmbH Heidelberg
			**Test name**	Acro COVID-19 Antigen Rapid Test	SARS-CoV-2 Antigen Test Kit (Immunochromatography)	New Coronavirus (SARS-CoV-2) N Protein Detection Kit (Fluorescence Immunchromatography)	CerTest Biotec SARS-CoV-2 Ag Test	COVID-19 Ag Respi-Strip	COVID-19 AG AllTest	Lumiratek SARS-CoV-2 Antigen Rapid Test Cassette	KaiBiLi COVID-19 Antigen Rapid Test Device	Novel Coronavirus (SARS-Cov-2) Antigen Rapid Test Cassette (swab)	Biozek medical COVID-19 Antigen Rapid Test Cassette	COVID-19 Antigen Rapid Test (Latex)	Joysbio SARS-CoV-2 Antigen Rapid Test Kit (Colloidal Gold)	Lionex COVID-19 Ag Rapid Test	Trueline COVID-19 Ag Rapid Test	QuickTestCorona COVID-19 Antigen Schnelltest
**Panel 1 V1**		**Panel 1 V2**		Panel 1 V1	Panel 1 V2	Panel 1 V2	Panel 1 V2	Panel 1 V1	Panel 1 V1	Panel 1 V2	Panel 1 V2	Panel 1 V2	Panel 1 V2	Panel 1 V2	Panel 1 V2	Panel 1 V2	Panel 1 V2	Panel 1 V2
**Pool Nr.**	**Cq**	**Pool Nr.**	**Cq**															
1	17,55	1	17,31	1	1	0	1	1	1	1	1	1	1	background	1	background	1	1
6	20,20	2	19,08	0	1	0	1	1	0	1	1	1	1	background	1	background	1	1
5	20,28	3	19,62	0	0	0	0	1	0	1	1	1	1	background	1	background	1	1
3	20,38	5	20,60	0	0	0	1	1	1	1	1	1	1	background	1	background	1	1
2	20,54	4	20,61	0	0	0	1	1	1	1	1	1	1	background	1	background	1	1
4	20,98	6	21,21	0	0	0	1	1	0	0	1	1	1	background	1	background	1	1
7	21,71	12	22,12	0	0	0	0	0	0	0	1	1	0	background	1	background	0	0
12	21,82	7	22,15	0	0	0	0	0	0	0	0	1	1	background	0	background	1	1
8	21,95	8	22,32	0	0	0	0	0	0	0	1	0	1	background	1	background	0	0
9	22,14	16	22,88	1	0	0	0	0	0	0	1	0	0	background	0	background	1	0
11	22,34	9	23,13	0	0	0	0	0	0	0	0	1	1	background	0	background	1	1
16	22,55	11	23,13	0	0	0	0	0	0	0	0	0	0	background	0	background	1	0
10	22,88	10	23,21	1	0	0	0	0	0	0	0	1	0	background	0	background	0	0
13	23,32	15	24,38	0	0	0	0	0	0	0	0	0	0	background	0	background	0	0
17	24,00	23	24,45	0	0	0	0	0	0	0	0	0	0	background	0	background	0	0
23	24,04	17	24,81	0	0	0	0	0	0	0	0	0	0	background	0	background	0	0
15	24,14	14	24,97	0	0	0	0	0	0	0	0	0	0	background	0	background	0	1
14	24,28	25	25,07	0	0	0	0	0	0	0	0	0	0	background	0	background	1	0
27	25,14	24	25,20	0	0	0	0	0	0	0	0	0	0	background	0	background	0	0
24	25,24	13	25,29	0	0	0	0	0	0	0	0	0	0	background	0	background	0	0
31	25,27	19	25,45	0	0	0	0	0	0	0	0	0	0	background	0	background	0	0
18	25,30	21	25,95	0	0	0	0	0	0	0	0	0	0	background	0	background	0	0
26	25,47	27	26,12	0	0	0	0	0	0	0	0	0	0	background	0	background	0	0
19	25,50	31	26,24	0	0	0	0	0	0	0	0	0	0	background	1	background	0	0
21	25,54	26	26,32	0	0	0	0	0	0	0	0	0	0	background	0	background	0	0
22	25,87	32	26,64	0	0	0	0	0	0	0	0	0	0	background	0	background	0	0
20	26,27	35	26,66	0	0	0	0	0	0	0	0	0	0	background	0	background	0	0
32	26,44	36	27,05	0	0	0	0	0	0	0	0	0	0	background	0	background	0	0
35	27,04	30	27,24	0	0	0	0	0	0	0	0	0	0	background	0	background	0	0
28	27,14	29	27,34	0	0	0	0	0	0	0	0	0	0	background	0	background	0	0
29	27,15	28	27,41	0	0	0	0	0	0	0	0	0	0	background	0	background	0	0
40	27,65	22	27,42	0	0	0	0	0	0	0	0	0	0	background	0	background	0	0
34	27,89	34	27,82	0	0	0	0	0	0	0	0	0	0	background	0	background	0	0
36	28,13	40	28,19	0	0	0	0	0	0	0	0	0	0	background	0	background	0	0
38	28,14	18	28,33	0	0	0	0	0	0	0	0	0	0	background	0	background	0	0
42	28,43	33	28,92	0	0	0	0	0	0	0	0	0	0	background	0	background	0	0
30	28,86	38	29,36	0	0	0	0	0	0	0	0	0	0	background	0	background	0	0
33	28,96	20	29,46	0	0	0	0	0	0	0	0	0	0	background	0	background	0	0
44	29,24	42	29,48	0	0	0	0	0	0	0	0	0	0	background	0	background	0	0
25	29,70	44	29,51	0	0	0	0	0	0	0	0	0	0	background	0	background	0	1
39	29,76	39	30,12	0	0	0	0	0	0	0	0	0	0	background	0	background	0	0
45	30,10	37	30,13	0	0	0	0	0	0	0	0	0	0	background	0	background	0	0
41	30,13	41	30,14	0	0	0	0	0	0	0	0	0	0	background	0	background	0	0
37	30,54	45	31,19	0	0	0	0	0	0	0	0	0	0	background	0	background	0	0
43	31,05	48	31,19	0	0	0	0	0	0	0	0	0	0	background	0	background	0	0
46	31,54	46	31,34	0	0	0	0	0	0	0	0	0	0	background	0	background	0	0
48	32,06	43	31,61	0	0	0	0	0	0	0	0	0	0	background	0	background	0	0
47	35,19	47	34,55	0	0	0	0	0	0	0	0	0	0	background	0	background	0	0
49	35,22	50	35,83	0	0	0	0	0	0	0	0	0	0	background	0	background	0	0
50	36,36	49	36,04	0	0	0	0	0	0	0	0	0	0	background	0	background	0	0

				112	113	114	115	116	117	118	119	120	121	122
			**Manufacturer**	nal von minden GmbH	Rapigen	Servoprax	Spring Healthcare Services SP zoo	SureScreen Diagnostics Ltd	TaiDoc Technology Corp.	Unioninvest	VivaChek Biotech(Hangzhou) Co,Ltd.	VivaChek Biotech(Hangzhou) Co,Ltd.	W.H.P.M, Inc	Xiamen Zhongsheng Langjie Biotechnology Co., Ltd
			**Test name**	dedicio Medical Test COVID-19 Ag plus Test	Biocredit COVID-19 Ag	Cleartest Coronaantigen	SARS-Cov-2 Antigen Rapid Test Cassette (swab)	COVID-19 Antigen Rapid Test Cassette	FORA COVID-19 ANTIGEN RAPID TEST	Unibioscience COVID-19 Rapid Antigen Test	VivaDiag SARS-CoV-2 Ag Rapid Test	VivaDiag Pro SARS-CoV-2 Ag Rapid Test	First SIGN SARS-CoV-2 Antigen Test	Covid-19 Antigen Test Cassette
**Panel 1 V1**		**Panel 1 V2**		Panel 1 V2	Panel 1 V1	Panel 1 V1	Panel 1 V2	Panel 1 V2	Panel 1 V1	Panel 1 V1	Panel 1 V1	Panel 1 V2	Panel 1 V2	Panel 1 V2
**Pool Nr.**	**Cq**	**Pool Nr.**	**Cq**											
1	17,55	1	17,31	1	1	1	1	1	1	0	1	1	1	1
6	20,20	2	19,08	1	0	1	1	1	1	0	1	1	1	1
5	20,28	3	19,62	1	1	1	1	1	1	0	1	1	1	0
3	20,38	5	20,60	1	1	1	1	1	1	0	1	1	1	0
2	20,54	4	20,61	1	0	1	1	1	1	0	1	1	1	0
4	20,98	6	21,21	0	0	1	0	1	0	0	1	1	1	0
7	21,71	12	22,12	0	0	1	0	1	0	0	1	1	0	0
12	21,82	7	22,15	0	0	1	0	1	0	0	1	1	0	0
8	21,95	8	22,32	0	0	1	0	1	0	0	0	1	1	0
9	22,14	16	22,88	1	0	1	0	0	0	0	1	0	0	0
11	22,34	9	23,13	0	0	0	0	0	0	0	0	1	1	0
16	22,55	11	23,13	0	0	0	0	0	0	0	0	0	0	0
10	22,88	10	23,21	0	0	1	0	0	0	0	0	1	0	0
13	23,32	15	24,38	0	0	1	0	0	0	0	0	0	0	0
17	24,00	23	24,45	0	0	0	0	0	0	0	0	0	0	0
23	24,04	17	24,81	0	0	0	0	0	0	0	0	0	0	0
15	24,14	14	24,97	0	0	0	0	0	0	0	0	0	0	0
14	24,28	25	25,07	0	0	0	0	0	0	0	0	0	0	0
27	25,14	24	25,20	0	0	0	0	0	0	0	0	0	0	0
24	25,24	13	25,29	0	0	0	0	0	0	0	0	0	0	0
31	25,27	19	25,45	0	0	0	0	0	0	0	0	0	0	0
18	25,30	21	25,95	0	0	0	0	0	0	0	0	0	0	0
26	25,47	27	26,12	0	0	0	0	0	0	0	0	0	0	0
19	25,50	31	26,24	0	0	0	0	0	0	0	0	0	0	0
21	25,54	26	26,32	0	0	0	0	0	0	0	0	0	0	0
22	25,87	32	26,64	0	0	0	0	0	0	0	0	0	0	0
20	26,27	35	26,66	0	0	0	0	0	0	0	0	0	0	0
32	26,44	36	27,05	0	0	0	0	0	0	0	0	0	0	0
35	27,04	30	27,24	0	0	0	0	0	0	0	0	0	0	0
28	27,14	29	27,34	0	0	0	0	0	0	0	0	0	0	0
29	27,15	28	27,41	0	0	0	0	0	0	0	0	0	0	0
40	27,65	22	27,42	0	0	0	0	0	0	0	0	0	0	0
34	27,89	34	27,82	0	0	0	0	0	0	0	0	0	0	0
36	28,13	40	28,19	0	0	0	0	0	0	0	0	0	0	0
38	28,14	18	28,33	0	0	0	0	0	0	0	0	0	0	0
42	28,43	33	28,92	0	0	0	0	0	0	0	0	0	0	0
30	28,86	38	29,36	0	0	0	0	0	0	0	0	0	0	0
33	28,96	20	29,46	0	0	0	0	0	0	0	0	0	0	0
44	29,24	42	29,48	0	0	0	0	0	0	0	0	0	0	0
25	29,70	44	29,51	0	0	0	0	0	0	0	0	0	0	0
39	29,76	39	30,12	0	0	0	0	0	0	0	0	0	0	0
45	30,10	37	30,13	0	0	0	0	0	0	0	0	0	0	0
41	30,13	41	30,14	0	0	0	0	0	0	0	0	0	0	0
37	30,54	45	31,19	0	0	0	0	0	0	0	0	0	0	0
43	31,05	48	31,19	0	0	0	0	0	0	0	0	0	0	0
46	31,54	46	31,34	0	0	0	0	0	0	0	0	0	0	0
48	32,06	43	31,61	0	0	0	0	0	0	0	0	0	0	0
47	35,19	47	34,55	0	0	0	0	0	0	0	0	0	0	0
49	35,22	50	35,83	0	0	0	0	0	0	0	0	0	0	0
50	36,36	49	36,04	0	0	0	0	0	0	0	0	0	0	0
